# Physical activity at work may not be health enhancing. A systematic review with meta-analysis on the association between occupational physical activity and cardiovascular disease mortality covering 23 studies with 655 892 participants

**DOI:** 10.5271/sjweh.3993

**Published:** 2022-02-25

**Authors:** Bart Cillekens, Maaike A Huysmans, Andreas Holtermann, Willem van Mechelen, Leon Straker, Niklas Krause, Allard J van der Beek, Pieter Coenen

**Affiliations:** 1 Department of Public and Occupational Health, Amsterdam UMC, Vrije Universiteit Amsterdam, Amsterdam Public Health Research Institute, Amsterdam, The Netherlands; 2 National Research Centre for the Working Environment, Copenhagen, Denmark; 3 Department of Sport Science and Clinical Biomechanics, University of Southern Denmark, Odense, Denmark; 4 School of Physiotherapy and Exercise Science, Curtin University, Perth, Australia; 5 Department of Environmental Health Sciences and Department of Epidemiology, School of Public Health, University of California Los Angeles, Los Angeles, CA, USA

**Keywords:** cardiovascular mortality, CVD

## Abstract

**Objectives:**

Emerging evidence suggests contrasting health effects for leisure-time and occupational physical activity. In this systematic review, we synthesized and described the epidemiological evidence regarding the association between occupational physical activity and cardiovascular disease (CVD) mortality.

**Methods:**

A literature search was performed in PubMed, Embase, CINAHL, PsycINFO and Evidence-Based Medicine Reviews, from database inception to 17 April 2020. Articles were included if they described original observational prospective research, assessing the association between occupational physical activity and CVD mortality among adult workers. Reviews were included if they controlled for age and gender and at least one other relevant variable. We performed meta-analyses on the associations between occupational physical activity and CVD mortality.

**Results:**

We screened 3345 unique articles, and 31 articles (from 23 studies) were described in this review. In the meta-analysis, occupational physical activity showed no significant association with overall CVD mortality for both males [hazard ratio (HR) 1.00, 95% confidence interval (CI) 0.87–1.15] and females (HR 0.95, 95% CI 0.82–1.09). Additional analysis showed that higher levels of occupational physical activity were non-significantly associated with a 15% increase in studies reporting on the outcome ischemic heart disease mortality (HR 1.15, 95% CI 0.88–1.49).

**Conclusions:**

While the beneficial association between leisure-time physical activity and CVD mortality has been widely documented, occupational physical activity was not found to have a beneficial association with CVD mortality. This observation may have implications for our appreciation of the association between physical activity and health for workers in physically demanding jobs, as occupational physical activity may not be health enhancing.

Physical activity plays an important role in the prevention of cardiovascular disease (CVD) (1, 2). Physical inactivity accounts for 7% of the global health burden (3) and is accompanied by considerable economic costs (4). Until recently, the health effects associated with work and leisure-time physical activity were considered beneficial and alike, as shown in a meta-analysis on all-cause mortality of cohort studies published until 2010 (5).

Evidence for the beneficial health effects of physical activity has mostly been derived from studies addressing leisure-time physical activity, exemplified in a meta-analysis with 44 studies reporting a 27% reduction of cardiovascular mortality risk for people with high compared to low-intensity leisure-time physical activity (6). However, recent evidence suggests contrasting health effects for leisure-time and occupational physical activity (7, 8). Specifically, while beneficial health outcomes have been reported for high levels of leisure-time physical activity, detrimental health consequences have been observed for high levels of occupational physical activity (9–11). A recent systematic review showed that males with high occupational physical activity had an 18% increased mortality risk, compared to those engaging in low occupational physical activity (12). Although no clear (negative or positive) associations were observed among females, these findings suggest that we may need to revise the way we look at physical activity, as not all domains of physical activity may be health enhancing.

One explanation for domain-specific health effects from physical activity may be the differences in acute and chronic physiological cardio-respiratory responses to physical activity, since domains differ substantially regarding their physical activity frequency, intensity, type, duration and recovery time (13). In contrast to leisure-time physical activity, occupational physical activity is of low intensity and long duration typically below the physiological threshold for improvement or maintenance of cardiovascular health (8). For example, in a sample of cleaners, who were highly active at work, occupational physical activity levels did not reach the intensity levels needed for cardiorespiratory fitness improvements (14). Even more so, the moderate occupational physical activity intensity was maintained over a long duration, and probably with insufficient time to recover, which can lead to chronic fatigue and prolonged elevated resting blood pressure (11) and heart rate (15, 16). These are established hemodynamic risk factors for atherosclerosis (17) and CVD (18, 19). Together, these findings suggest that the potential differential mortality effects of occupational and leisure-time physical activity may be driven by cardiovascular mechanisms (13). It is therefore important to study the cardiovascular health consequences of occupational physical activity, in particular because CVD is the leading cause of death worldwide (20). Although these hypothesized mechanisms hold mainly for occupations involving manual materials handling, prolonged working postures and/or prolonged activity, they may not apply to all jobs with high level occupational physical activity (eg, elite athletes) (12).

A recent review showed a linear beneficial effect of leisure-time physical activity on CVD mortality (6). However, reviews on the associations between occupational physical activity and CVD have been inconclusive and conflicting and the most recent meta-analysis has been published in 2013. For instance, a review of 11 studies published until 2001 showed that being active at work was associated with a lower risk for stroke (21), whereas an earlier review showed no effect of occupational physical activity on hypertension risk (22). A later review included only four cohort studies with occupational physical activity measures and showed a protective effect of high (versus low) occupational physical activity on CVD (23). However, the most recently published meta-analyses of seven cohort studies suggested that high (versus low) levels of occupational physical activity are associated with 24% and 25% increased risk of ischemic heart disease (IHD) and overall CVD, respectively (7).

Although these reviews examined the association between occupational physical activity and CVD risk, none of them specifically examined the association of occupational physical activity with CVD mortality. Therefore, the aim of our study was to systematically review the epidemiological evidence regarding the association between occupational physical activity and CVD mortality from prospective cohort studies.

## Methods

### Search for literature

This review was a priori registered (PROSPERO) (24) and executed according to the PRISMA guidelines (25). Systematic searches were performed in bibliographic databases PubMed, Embase, CINAHL, PsycINFO and the Cochrane Central Register of Controlled Trials, from database inception to 17 April 2020 using controlled key and free text search terms expressing physical activity, occupational and mortality (supplementary material, www.sjweh.fi/article/3993, table S1). Reference lists of included articles were screened for additional studies.

Two reviewers independently screened all potentially relevant titles and abstracts for eligibility and, if necessary, full-text articles. In case of disagreement, consensus was reached by consulting a third reviewer. Articles were included if they met the following criteria: English language, original peer-reviewed prospective cohort studies assessing the association between occupational physical activity and CVD mortality in adult workers selected from a general working population. Studies assessing occupational physical activity directly through self-report or wearable sensors (such as accelerometers or heart rate monitors) were included. Studies assessing occupational physical activity indirectly by task and/or job classification (eg, blue- versus white-collar workers, or manual versus non-manual work) were excluded. Studies on occupational sedentary behavior (rather than physical activity) were only included in cases with relevant reference groups engaging in at least moderate level occupational physical activity (excluding studies assessing various durations of sedentary behaviors).

Consistent with our previous review (12), we included only studies controlling for (either by adjustment or stratification) age, gender and at least one other potentially confounding factor, such as psychosocial job factors (eg, job stress, supervisor support, or shift work), socio-economic status (eg, education or income), lifestyle (eg, smoking, alcohol consumption or leisure-time physical activity), or biological factors (eg, body mass index, serum lipids, diabetes, or pre-existing cardiovascular conditions). In case there were more articles on the same study, then the article describing the longest follow-up period was used in the meta-analysis.

### Data extraction and risk of bias assessment

Two reviewers independently extracted data and assessed risk of bias. In cases of disagreement, consensus was reached by consulting a third reviewer. Extracted data included: first author, publication year, study name and design, follow-up period, sample characteristics, assessment methods for mortality outcomes and occupational physical activity, effect estimates and adjustments. Corresponding authors were asked for additional information if needed.

Risk of bias was scored by 12 criteria related to reporting of study methods and results (supplementary table S2) (26). Summary scores >75% indicated high methodological quality, hence low risk of bias.

### Data analysis

During consensus meetings, various differently defined occupational physical activity categories from different studies were harmonized by classifying them into one of four categories on the physical activity continuum: (i) occupational sedentary behavior, (ii) low level occupational physical activity, (iii) moderate level occupational physical activity, and (iv) high level occupational physical activity (supplementary table S3). Although the categories differ substantially, they roughly depict: (i) mainly sitting work (sedentary), (ii) mostly standing some walking (low), (iii) standing/walking and/or carrying light objects (moderate), and (iv) physically demanding work (high).

Participants across study samples were pooled according to their assigned harmonized exposure categories. Occupational physical activity effects on mortality were estimated as hazard ratios (HR) with 95% confidence intervals (CI) derived from inverse variance random effects models (27), comparing the highest harmonized occupational physical activity category to the lowest. Sensitivity analyses investigated different effects contrasting sedentary or low levels of occupational physical activity with moderate and high levels of occupational physical activity.

Analyses were performed using Review Manager [RevMan, V.5.3 (computer program)]. Forest plots depicted individual study and pooled effect estimates. Heterogeneity was assessed by visual inspection of forest plots and I^2^statistics (I^2^>50% indicating substantial heterogeneity) (28). Funnel plots were generated in R studio (R Development Core Team, 2015) to assess publication bias using packages ’robumeta’, ’metafor’ and ’dplyr’ (29). Rank correlation (30) and regression tests (31) were used to calculate potential bias. Sensitivity analysis examined whether any study influenced the pooled effect size (>10%) by excluding one study at a time from the analysis pool.

As stipulated in our a priori registered protocol (24), results were stratified by gender, mortality type (CVD in general or IHD), occupational physical activity measurement (quantitative versus qualitative) and adjustment (for other domains of physical activity/socio-economic status/diet and body mass index). We additionally stratified for sample size (< or >10 000), baseline examination (before or after median examination date) and follow-up duration (categorizing studies using tertiles according to follow-up duration).

## Results

### Data Sources

**Figure 1:**
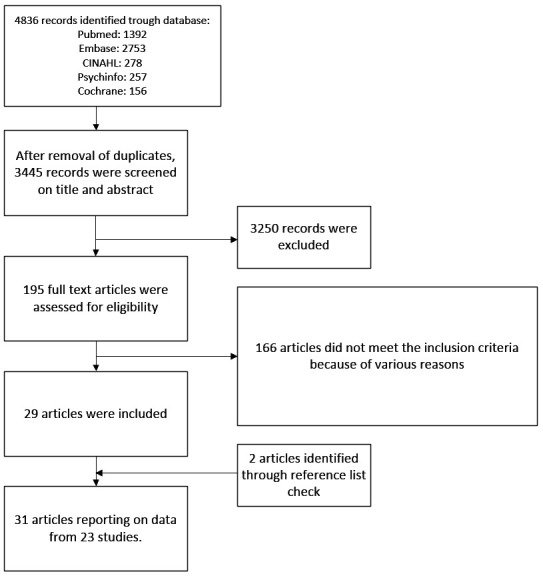
Flowchart depicting the search and selection procedure.

The literature search generated 3445 unique references (figure 1). After screening titles and abstracts 195 full text articles were retrieved; 166 of them were excluded for various reasons (supplementary table S4). Adding two papers from reference lists yielded 31 articles from 23 studies for our review (32–52) (supplementary table S5). Two articles described two study populations each (49, 52).

Supplementary table S6 summarizes descriptive data of reviewed studies. Five of the seven contacted authors provided additional data (33, 38, 50–52). Ten studies examined each gender separately, eight were limited to males, and five examined a mixed population. Occupational physical activity was assessed by self-report between 1960 and 2004, mortality by death registries as overall CVD mortality in sixteen studies, IHD in four, and three examined both. Nineteen studies originated from Europe, three from Asia and one from Australia. Nine of the nineteen European studies were from Nordic countries (Norway, Sweden, Denmark, Finland).

The average methodological quality was 90%, (range 71–100%), with 20 articles classified as low risk of bias (>75%; supplementary table S7). Important sources of risk of bias were participant selection, exposure assessment, controlling for confounding and analysis method.

### Meta-analysis

The selected 23 studies included 655 892 participants with over 8.2 million person-years in follow-up. Pooled analyses contrasting highest versus lowest occupational physical activity levels indicated no association with overall CVD (HR 0.99, 95% CI 0.90–1.09, I^2^=71%; table 1) (Forest plot; supplementary figure S2), neither for males (HR 1.00, 95% CI 0.87–1.15) nor females (HR= 0.95, 95% CI 0.82–1.09). Effect estimates showed substantial heterogeneity (I^2^ ≥50%). Funnel plots, rank correlation test (z=1.19, P=0.14), and Egger’s regression test (Kendall’s tau=0.18, P=0.23) all indicated low risks of publication bias (supplementary figure S1). High levels of occupational physical activity were associated with statistically non-significant, 15% increased risk of IHD (HR 1.15, 95% CI 0.88–1.49).

**Table 1:** Meta-analyses depicting the association between occupational physical activity (highest versus lowest) and cardiovascular disease mortality. Comparisons from 23 studies with 655 892 participants are depicted in the upper row. Associations are shown stratified by gender, type of mortality, occupational physical activity measurement, follow-up period, year of baseline assessment, sample size of study, adjusting for confounders. [HR=hazard ratio; CI=confidence interval; I^2^=heterogeneity; LTPA=leisure time physical activity; SES=social economic status; BMI=body mass index]

Increments of occupational physical activity	Studies	N ^a^	HR	95% CI	I^2^%
Highest vs lowest occupational physical activity category	23	33	0.99	0.90–1.09	71
Males	18	18	1.00	0.87–1.15	80
Females	10	10	0.95	0.82–1.09	35
Type of mortality					
Overall cardiovascular mortality ^b^	19	29	0.95	0.86–1.05	67
Ischemic heart disease mortality ^b^	7	9	1.15	0.88–1.49	60
Occupational physical activity measurement					
Quantitative self-rated measurements (eg, amount of stairs/hours/frequency)	6	8	0.92	0.70–1.20	65
Qualitative self-rated measurements (eg, non quantifiable exposure or exertion)	17	25	1.01	0.91–1.12	73
Follow up duration (years)					
3.3–13.6	8	10	1.02	0.78–1.34	74
15.9–21.7	7	11	0.92	0.80–1.07	71
22–50	8	12	1.11	0.93–1.33	74
Year of baseline assessment					
Before 1989	12	17	0.96	0.87–1.07	61
1990 and later	11	16	1.03	0.83–1.27	77
Sample size of study					
≤10 000	15	20	1.09	0.91–1.30	67
>10 000	8	13	0.88 ^c^	0.80–0.97	77
Adjusted for total or leisure-time physical activity.					
No	5	6	0.98	0.76–1.26	80
Yes	16	27	1.01	0.91–1.13	70
Adjusted for socioeconomic status (education or social class)					
No	6	6	0.89	0.67–1.17	79
Yes	17	27	1.01	0.90–1.14	76
Adjusted for BMI (or waist circumference)					
No	4	6	1.12	0.86–1.47	79
Yes	19	27	0.95	0.86–1.06	68
Adjustment for diet (Energy intake/ Mediterranean diet)					
No	19	27	1.01	0.91–1.13	76
Yes	4	6	0.84	0.70–1.00	0
Adjusted for gender, age, smoking, LTPA, SES and BMI					
No	9	11	1.00	0.83–1.22	80
Yes	14	22	0.97	0.86–1.08	64

a Number of estimates, which could be more than the number of studies because separate estimates were reported for males and females.

b International Classification of Diseases and Related Health Problems codes are reported in supplementary table S6. References for all estimates provided in supplementary table S8.

c Statistically significant.

High occupational physical activity was associated with an 11% increase of overall CVD mortality (HR 1.11, 95% CI 0.93–1.33) in studies with ≥22 follow-up years and a 12% decrease in studies with larger sample sizes (HR 0.88, 95% CI 0.80–0.97). Year of baseline assessment, adjustment for leisure-time physical activity, or adjustment for gender, age, socio-economic status (SES) together did not influence the results. However, studies adjusting for body mass index (BMI) or diet showed lower effect sizes. Exclusion of any study from the pooled data did not substantially influence effect size or heterogeneity.

Sensitivity analyses using different reference groups showed no effect changes between occupational physical activity and CVD mortality, nor did comparing moderate and low occupational physical activity with sedentary occupational physical activity (table 2). Comparisons based on the moderate rather than high occupational physical activity group showed differential effects, with a lower CVD risk estimate for moderate occupational physical activity versus low occupational physical activity (HR 0.85, 95% CI 0.77–0.94), compared to moderate versus sedentary (HR 1.03, 95% CI 0.92–1.15).

**Table 2:** Sensitivity analyses. Meta-analyses depicting the association between occupational physical activity groups and cardiovascular disease mortality adjusting for confounders (at least age, gender and smoking and additional variables). [HR=hazard ratio; CI=confidence interval; I^2^=heterogeneity]

Comparisons	Studies	N ^a^	HR	95% CI	I^2^%
High versus low	12	15	0.98	0.82–1.16	64
Males	8	8	0.93	0.73–1.19	74
Females	4	4	1.06	0.67–1.66	49
High versus sedentary	9	13	1.02	0.84–1.22	86
Males	8	8	1.06	0.82–1.37	85
Females	4	4	1.03	0.81–1.30	45
Moderate versus low	4	7	0.85 ^b^	0.77–0.94	39
Males	3	3	0.87	0.74–1.03	64
Females	3	3	0.75 ^b^	0.63–0.89	0
Moderate versus sedentary	10	15	1.03	0.92–1.15	79
Males	8	8	1.09	0.95–1.25	66
Females	5	5	1.02	0.89–1.16	20
Low versus sedentary	7	11	0.95	0.87–1.05	72
Males	5	5	1.02	0.86–1.21	60
Females	4	4	1.02	0.87–1.18	26

a Number of estimates, which could be more than the number of studies because separate estimates were reported for males and females.

b Statistically significant.

## Discussion

Our meta-analyses of 655 892 participants with over 8.2 million person-years follow-up showed that occupational physical activity was not related to overall CVD mortality. These findings suggest that the beneficial health effects of physical activity that are frequently reported (6) and generally accepted for leisure-time physical activity may not apply to occupational physical activity with regards to CVD mortality. Further, given the non-significant 15% increase in IHD mortality risk, high occupational physical activity may even lead to detrimental effects. This opposing effect may require a revision of the way we look at physical activity and health for workers in jobs with high occupational physical activity because most physical activity guidelines do not differentiate between domains of physical activity.

For a considerable fraction of the adult population, occupational physical activity constitutes the main portion of their overall daily physical activity (8, 53). These workers could potentially (easily) achieve the recommended amounts, without engaging in any type or amount of leisure-time physical activity. Workers who are highly active at work are known to be fairly inactive during their leisure-time (54), and they might not benefit from the work domain of physical activity in a way that has been observed for leisure-time physical activity (8). Exhaustion due to long work periods without sufficient breaks and recovery periods may be a reason that high volumes of occupational physical activity are negatively associated with leisure-time physical activity. The differential health effects of occupational and leisure-time physical activity, in the literature referred to as the ’physical activity paradox’ (55), are suggested to be due to aforementioned physiological differences in the nature of the activity (12, 13).

There are conceptual differences between occupational and leisure-time physical activity. From an occupational health standpoint, occupational physical activity is often defined as consisting of exposure to demanding working postures, repetitive movements and manual materials handling. In public health, physical activity is more often captured by intensity, frequency and duration of aerobic activities and/or energy expenditure. This indicates the different conceptual ways in which physical activity is considered in both research fields. Future research should move beyond these differences and incorporate all aspects of occupational physical activity to get a better understanding of its health effects (56). In the supplementary text A, we elaborate on whether or not this review supports the physical activity paradox.

### Comparison with previous literature reviews

This is the first review to systematically synthesize the evidence on occupational physical activity and CVD mortality. Other reviews examined the relationship between leisure-time physical activity or total physical activity, and CVD mortality and found higher levels of these physical activity domains to be associated with lower risk of CVD mortality (6, 57–59). A review of seven studies published between 2011 and March 2013 showed an inverse association of leisure-time physical activity and a positive association of occupational physical activity with overall CVD [risk ratio (RR) 1.24, 95% CI 1.05–1.47) and IHD incidence (RR 1.25, 95% CI 1.05–1.51; six out of seven studies) (7). In an earlier review (60) of three studies published between 1980 and 2010, the same authors had reported a protective effect of occupational physical activity on CVD incidence (RR 0.77, 95% CI 0.77–0.92). Our review differs from these reviews by type (mortality versus incidence) and specificity of CVD outcome (mostly overall CVD versus mostly IHD) and our null finding for overall CVD mortality risk. Our findings of a 15% increase in IHD mortality risk related to high occupational physical activity is consistent with the 25% IHD incidence risk reported in the most recent review by Li (7).

Results from our previous meta-analysis showed that men with high occupational physical activity had an 18% increased risk of all-cause mortality (12). A discussion paper suggested that the effects of occupational physical activity on all-cause mortality may be mostly driven by CVD mechanisms (13). This latter hypothesis, however, was not affirmed in our current study on CVD mortality. A possible explanation for this could be that effects regarding the association between occupational physical activity and all-cause mortality for men may not be solely the result of CVD mechanisms.

The increased risk of all-cause mortality may potentially be caused by other agents and diseases that may co-occur with high levels of occupational physical activity, such as exposure to occupational carcinogens or radiation. A recent review reported a 15% increased risk of lung cancer in men with high occupational physical activity after adjustment for age and smoking (61). Exposure to various air pollutants at work may increase both cardiovascular, all-cause and cancer mortality among industrial workers with high occupational physical activity levels from low-income blue-collar communities. Work-related fatal injuries or non-cardiovascular illness may be another pathway for occupational physical activity towards increased all-cause mortality (12, 62).

The results could partly be explained by IHD mortality which showed a non-significant, 15% increase (HR 1.15, 95% CI 0.88–1.49). Most evidence about differential health effect of occupational and leisure-time physical activity are based on differentials in IHD risk (8, 13), which is understood to be the result of arteriosclerotic changes caused by CVD risk factors such as heart rate, blood pressure and associated inflammatory changes (55). The overall-CVD outcome measure is not limited to diseases caused by ischemia, but also include diseases that are caused by infection (eg, endocarditis, myocarditis) or those that are secondary to chronic pulmonary diseases that have not been hypothesized to be prevented or caused by any form of physical activity. Lumping those different diseases into a single outcome is likely to lead to misclassification bias, diluting the effect size of occupational physical activity compared to studies based solely on IHD or stroke, which share the same pathophysiological pathways that are thought to be affected by physical activity. If this hypothesis holds true, the increased risk of IHD mortality (which is part of overall CVD) and the null effect of overall CVD could indicate that other CVD outcomes show a beneficial effect.

Since 19 of our 23 reviewed studies included non-specific CVD outcomes, with diagnostic subgroups that have not hypothesized to be related to occupational physical activity, lower effect estimates for overall CVD compared to IHD are to be expected. In addition to this outcome misclassification bias, residual confounding, exposure misclassification, and health-based selection effects may contribute to inconsistencies and the high heterogeneity in our pooled findings (I^2^=76%).

### Residual confounding

Although most of the studies (61%) included in our review adjusted for at least gender, SES, age, leisure-time physical activity, smoking and BMI, residual confounding cannot be ruled out. It might be possible that unmeasured and/or residual confounding could lead to an overestimation of the presented findings, and therefore result in biased results (63). This notion is underlined in a recent publication in which the negative health effects of occupational physical activity presented in partially adjusted models, change to positive health effects when additionally adjusting for various socioeconomic variables (64). However, mutually adjusting for several measures of SES and their associated behavioral factors that are highly correlated with occupation physical activity, could also have methodological drawbacks and possibly lead to over adjusting (65). It might also be possible that the association between occupational physical activity and CVD outcomes is confounded by social and behavioral factors that occurred prior to entering the study or even the workforce (66).

It must be acknowledged that the current literature has not yet succeeded in identifying and evaluating all confounders, mediators and moderators in the relationship between occupational physical activity and health (56). Therefore, we recommend future research to use causal inference modelling and directed acyclic diagrams (DAG) on observational data to describe mechanistic pathways, or using alternative (eg, experimental) research designs that could address confounders, mediators and moderators in a better way (64).

We performed subgroup analyses comparing studies with and without controlling for key potential confounders to assess the potential impact of uncontrolled confounding for heterogeneity and observed effect sizes. Overall, different adjustments decreased heterogeneity very little and led to small changes (ie, increases or decreases) in effect sizes, indicating both negative and positive confounding, albeit of modest magnitude. Of note, studies adjusting for SES showed higher occupational physical activity risks for overall CVD mortality compared to unadjusted studies, suggesting negative confounding by SES and rendering mediation of occupational physical activity by uncontrolled SES-related factors unlikely, as has been discussed (63) and reported on in the literature (67). In contrast, adjustment for BMI or waist circumference or diet led to small reductions in occupational physical activity risks, indicating that these variables could be confounders, mediators or both.

Adjustment for leisure-time physical activity also resulted in a small increase of occupational physical activity risks on CVD mortality. This raises the concern that some of the evidence regarding beneficial effects of leisure-time physical activity may potentially be confounded by non-beneficial or even detrimental effects of occupational physical activity, since most studies on leisure-time physical activity did not adjust for occupational physical activity. Several studies that simultaneously analyzed or investigated interactions of leisure-time and occupational physical activity showed inconsistent results (40, 68–72).

Future research should therefore investigate what aspects of occupational physical activity as well as what combinations of occupational and leisure-time physical activity can be beneficial, not only for healthy young workers, but also for older workers and workers with pre-existing CVD or other comorbidities (73).

High occupational physical activity might also co-occur with other unmeasured occupational exposures, which may have a direct or indirect association with CVD disease and death, such as exposure to chemicals, environmental heat, noise, dust, air pollution and various psychosocial job stressors. It is possible that not controlling for these potentially modifying risks factors may have led to an over- or underestimation of the independent effect of occupational physical activity. Future research should evaluate these factors, not only as potential confounders but also as mediators and/or effect modifiers.

### Exposure misclassification

A major limitation of the reviewed evidence is the likelihood of non-differential exposure misclassification leading to an underestimation of reported effect sizes. Non-differential exposure misclassification occurred not only in all reviewed original studies but additionally in our meta-analyses of these studies where we classified occupational physical activity dichotomously (74).

All studies used self-report measures of occupational physical activity; some studies were limited to indicators of strenuous activities (eg, heavy lifting, climbing stairs, sweating, tiredness after work), others focused on body postures or movements (eg, sitting, standing, walking), or calculated absolute measures of energy expenditure (eg, metabolic equivalent of tasks (hours/week, kcal/day). Only one study used relative aerobic strain measures, taking individual levels of cardiorespiratory fitness into account which is relevant for the actual strain on the cardiovascular system (40). Specifically, occupational physical activity measured as relative aerobic strain [ie, energy expenditure at work relative to maximal oxygen uptake (%VO_2_max)] yielded 13% and 21% higher all-cause and IHD mortality risks, respectively, than occupational physical activity expressed as absolute energy expenditure at work (kcal/workday) (40). Similar differences were reported for IHD incidence in the same study population (70). This indicates that ignoring the individual workers maximal oxygen uptake capacity (ie, the actual aerobic work capacity) can lead to substantive conservative bias, as was demonstrated in these studies were effect estimates based on absolute measures of workload were less predictive of risk. In our meta-analyses, we did not find a substantial difference between studies that used more quantitative self-rated measurements (eg, amount of stairs/hours/frequency) compared to studies that used more qualitative measurements (eg, sitting, standing, walking) (table 1).

Most studies used crude dichotomous measures or collapsed continuous measures of occupational physical activity into broad categories, typically defined as population frequencies (eg, sample tertiles or quartiles). Combining highest occupational physical activity intensities with much lower intensities could lead to a dilution of the effects, especially among women who compared to men are less frequently working at the highest occupational physical activity categories. This may explain why substantial effects of high occupational physical activity are mostly based on male cohorts and have rarely been described among females.

Choice of comparison categories and current job versus previous job may also determine results as reported in a recent large cohort study on occupational physical activity and stroke (75). The effect estimates of the lowest occupational physical activity category (mostly sitting) with the highest (heavy manual labor, HR 0.87, 95% CI 0.36–2.13) were smaller, more imprecise, and changed direction compared to the second-highest category (continuous walking/movement, HR 1.53, 95% CI 1.14–2.07). Comparing the longest held job with the estimates based on current job also showed a 11% difference in effect estimates.

In future studies, more sophisticated assessments of occupational physical activity less prone to misclassification, could lead to more accurate results. Future research should use physical activity measurements using heart rate monitors, accelerometers, inclinometers and/or other observational ergonomic methods to assess the repetitive or continuous duration and intensity of physical activity, work postures, relative aerobic workload, breaks, and recovery time (76).

Improved observational measures of occupational physical activity through modern sensor technology alone may not be sufficient to overcome other important sources of misclassification of occupational physical activity. It is also necessary to arrange repeated measurements during follow-up, preferably with work history and other relevant factors starting from baseline as well as factors before entering the workforce and to collect a combination of administrative, self-reported and expert-based data that can be utilized to construct a detailed job and health history, capturing changes in physical job demands, comorbidity, cardiovascular fitness, and important confounders and effect modifiers during follow-up. Future research also needs to determine precise dose–response relationships and explore threshold effects that can be used for the development for specific physical activity guidelines (77). Further, more studies need to use relative physical activity measures that account for workers physical capacities to guide the development of appropriate ergonomic work modifications, effective workplace health promotion exercise programs, and safe and effective clinical, rehabilitation, and public health physical activity guidelines.

### Selection bias

Another potential bias, which can cause inconsistencies in the literature regarding the cardiovascular health effects of occupational physical activity is due to the selection of healthy workers into physically demanding jobs and of diseased workers with activity-related symptoms (such as angina pectoris) moving into less strenuous jobs or leaving the workforce (70, 78). This deflates CVD risks among workers found in these demanding jobs at the time of the study (79) and could have led to an underestimation of the true occupational physical activity effect in our review. Future research should quantify the magnitude of such biases and account for it in effect estimation. Gathering complete life work histories is a prerequisite for accounting for changes in the nature of work and cumulative occupational physical activity exposures that are most relevant for the development of chronic diseases such as CVD and associated mortality outcomes.

Most methods proposed to address healthy worker biases, however, do not consider that it may be caused by time-varying confounders affected by prior exposure. G-estimation of accelerated failure-time models have been developed to handle this issue but has never been applied to account for the healthy-worker survivor effect in studies of occupational physical activity and mortality (80).

Comparing early stage non-symptomatic CVD outcomes such as sonographically measured progression of intima media thickness of arterial walls with symptomatic CVD disease outcomes such as CHD with activity-related angina pectoris in the same study population can also be used to identify the likelihood and potential extent of healthy worker biases as demonstrated by a series of Finnish cohort studies on progression of atherosclerosis (68, 81), acute myocardial infarction (70), and mortality (40).

### Strengths and limitations

Restriction to studies of high methodological quality including only prospective cohort studies with adjustment for key confounders, collection of additional unpublished data, pooling of data from 655 794 participants in a meta-analysis with additional subgroup analyses are methodological strengths of this review.

We mentioned the potential influence of residual confounding, exposure misclassification and health-based selection effects on our study results in the above. An additional limitation is that our meta-analysis was based nearly exclusively on high-income countries, and are not readily generalizable to low- and middle-income countries where occupational physical activity may constitute a higher proportion of the total physical activity (82). Although we prospectively registered mortality by stroke as a condition, we could not perform analyses because only one included study reported on this specific outcome (50).

Another limitation is that, although all eligible studies adjusted for age, we could not perform a subgroup analysis by age groups or physical fitness level. Although overall physical job demands in high-income countries may have been reduced through regulation of maximum regular work hours, mechanization, and global redistribution of manufacturing, the average absolute physical energy spent at work has remained rather stable for the individual aging worker, as has been demonstrated in one of the reviewed study cohorts, where energy expenditure on average decreased only by about 0.16% kcal per year over a four-year period (68), while cardiorespiratory fitness decreased 8.5 times faster by 1.36% per year (83). Cardiorespiratory fitness decreases with age (84), and therefore identical work tasks become relatively more strenuous for older workers. Ignoring the role of aerobic fitness in assessing the effect of physical workload on health may have led to a conservative bias in our current study and previous literature (12). The a-priory registered methodological quality scale we used,may retrospectively not be the best tool in detecting risk of bias (26), we recommend future reviews to use a more appropriate instrument.

### Concluding remarks

This meta-analyses of 23 prospective studies, with 655 892 participants, showed that higher occupational physical activity was not related with overall CVD mortality (HR 0.99, 95% CI 0.89–1.10), but was positively associated with IHD mortality risk (HR 1.15, 95% CI 0.88–1.49). The findings are mostly compatible with the hypothesis that high levels of occupational physical activity do not confer cardiovascular health benefits, in contrast to beneficial leisure-time physical activity effects often reported in the literature. Study design features, such as country, historical time and duration of follow-up period, had no substantial effect on the effect estimate. However, due to the lack of understanding on the complex role of socioeconomic status in the association of occupational physical activity and health, and the lack of measuring environmental occupational exposures, the risk of residual confounding cannot be ruled out based on the current evidence. The high likelihood of non-differential exposure misclassification of occupational physical activity and healthy worker effects most likely led to an underestimation of any effect of high occupational physical activity.

Future research should differentiate between different physical activity domains, reduce occupational physical activity exposure misclassification, and examine potential interactions with pre-existing CVD, cardiorespiratory fitness, leisure-time physical activity, SES, and other CVD risk factors in the working environment.

## Supplementary material

Supplementary material
