# Supplementary material for: Physical activity at work may not be health enhancing. A systematic review with meta-analysis on the association between occupational physical activity and cardiovascular disease mortality covering 23 studies with 655 892 participants
Source: Scand J Work Environ Health. 2022 Feb 25;48(2):86–98. doi: 10.5271/sjweh.3993 (PMC9045238; doi:10.5271/sjweh.3993)
Supplement: Supplementary material [file SJWEH-48-86-S001.pdf]

Physical activity at work may not be health enhancing. A systematic review with meta-analysis on the association between occupational physical activity and cardio-vascular disease mortality covering 23 studies with 655 892 participants[1]

by Bart Cillekens, MSc, Maaike A Huysmans, PhD, Andreas Holtermann, PhD, Willem van Mechelen, PhD, Leon Straker, PhD, Niklas Krause, PhD, Allard J van der Beek, PhD, Pieter Coenen, PhD [2]

1. *Supplementary material*
2. *Correspondence to: Dr. Pieter Coenen, Department of Public and Occupational Health, Amsterdam Public Health Research Institute, VU University Medical Centre (VUmc), Van der Boechorststraat 7, 1081 BT, Amsterdam, The Netherlands. [E-mail: p.coenen@amsterdamumc.nl]*

Table S1. Search strategies.

Search strategy in PubMed (read from bottom-up).

No.	Query
#5	#1 AND #2 AND #3 AND #4
#4	"Cohort Studies"[Mesh] OR cohort*[tiab] OR longitudinal[tiab] OR prospective[tiab] OR follow-up[tiab]
#3	"Mortality"[Mesh] OR "Longevity"[Mesh] OR mortality[tiab] OR longevity[tiab] OR death[tiab]
#2	"Employment"[Mesh] OR "Work"[Mesh] OR "Occupational Health"[Mesh] OR "Occupational Diseases"[Mesh] OR "Occupations"[Mesh] OR work[tiab] OR occupation*[tiab] OR vocation*[tiab] OR employ*[tiab] OR job[tiab] OR jobs[tiab]
#1	"Motor Activity"[Mesh:NoExp] OR "Exercise"[Mesh] OR "Physical Exertion"[Mesh] OR motor activit*[tiab] OR physical activit*[tiab] OR locomotor activit*[tiab] OR exercis*[tiab] OR training[tiab] OR stretching[tiab] OR physical condition*[tiab] OR physical fitness[tiab] OR physical endurance[tiab] OR physical demand[tiab] OR physical exposure[tiab] OR physical exertion[tiab]

Search strategy in Embase (read from bottom-up).

No.	Query
#5	#1 AND #2 AND #3 AND #4
#4	'cohort analysis'/exp OR cohort*:ab,ti OR longitudinal:ab,ti OR prospective:ab,ti OR 'follow-up':ab,ti
#3	'mortality'/exp OR 'longevity'/exp OR mortality:ab,ti OR longevity:ab,ti OR death:ab,ti
#2	'occupation'/exp OR 'work'/exp OR 'occupational health'/exp OR 'occupational disease'/exp OR work:ab,ti OR occupation*:ab,ti OR vocation*:ab,ti OR employ*:ab,ti OR job:ab,ti OR jobs:ab,ti
#1	'motor activity'/de OR 'exercise'/exp OR 'motor activit*':ab,ti OR 'physical activit*':ab,ti OR 'locomotor activit*':ab,ti OR exercis*:ab,ti OR training:ab,ti OR stretching:ab,ti OR 'physical condition*':ab,ti OR 'physical fitness':ab,ti OR 'physical endurance':ab,ti OR 'physical demand':ab,ti OR 'physical exposure':ab,ti OR 'physical exertion':ab,ti

Search strategy in CINAHL (read from bottom-up).

No.	Query
#5	#1 AND #2 AND #3 AND #4

#4	(MH "Prospective Studies+") OR TI (cohort* OR longitudinal OR prospective OR "follow-up") OR AB (cohort* OR longitudinal OR prospective OR "follow-up")
#3	(MH "Mortality+") OR (MH "Longevity") OR TI (mortality OR longevity OR death) OR AB (mortality OR longevity OR death)
#2	(MH "Employment+") OR (MH "Work") OR (MH "Occupational Health+") OR (MH "Occupational Diseases+") OR (MH "Occupations and Professions+") OR TI (work OR occupation* OR vocation* OR employ* OR job OR jobs) OR AB (work OR occupation* OR vocation* OR employ* OR job OR jobs)
#1	(MH "Motor Activity") OR (MH "Exercise+") OR TI ("motor activit*" OR "physical activit*" OR "locomotor activit*" OR exercis* OR training OR stretching OR "physical condition*" OR "physical fitness" OR "physical endurance" OR "physical demand" OR "physical exposure" OR "physical exertion") OR AB ("motor activit*" OR "physical activit*" OR "locomotor activit*" OR exercis* OR training OR stretching OR "physical condition*" OR "physical fitness" OR "physical endurance" OR "physical demand" OR "physical exposure" OR "physical exertion")

Search strategy in PsychINFO (read from bottom-up).

No.	Query
#5	#1 AND #2 AND #3 AND #4
#4	(MH "Prospective Studies+") OR TI (cohort* OR longitudinal OR prospective OR "follow-up") OR AB (cohort* OR longitudinal OR prospective OR "follow-up")
#3	(MH "Mortality+") OR (MH "Longevity") OR TI (mortality OR longevity OR death) OR AB (mortality OR longevity OR death)
#2	(MH "Employment+") OR (MH "Work") OR (MH "Occupational Health+") OR (MH "Occupational Diseases+") OR (MH "Occupations and Professions+") OR TI (work OR occupation* OR vocation* OR employ* OR job OR jobs) OR AB (work OR occupation* OR vocation* OR employ* OR job OR jobs)
#1	(MH "Motor Activity") OR (MH "Exercise+") OR TI ("motor activit*" OR "physical activit*" OR "locomotor activit*" OR exercis* OR training OR stretching OR "physical condition*" OR "physical fitness" OR "physical endurance" OR "physical demand" OR "physical exposure" OR "physical exertion") OR AB ("motor activit*" OR "physical activit*" OR "locomotor activit*" OR exercis* OR training OR stretching OR "physical condition*" OR "physical fitness" OR "physical endurance" OR "physical demand" OR "physical exposure" OR "physical exertion")

Search strategy in Cochrane (read from bottom-up).

No.	Query
#5	#1 AND #2 AND #3 AND #4
#4	(cohort* OR longitudinal OR prospective OR "follow-up"):ab,ti,kw
#3	(mortality OR longevity OR death):ab,ti,kw
#2	(work OR occupation* OR vocation* OR employ* OR job OR jobs):ab,ti,kw
#1	("motor activit*" OR "physical activit*" OR "locomotor activit*" OR exercis* OR training OR stretching OR "physical condition*" OR "physical fitness" OR "physical endurance" OR "physical demand" OR "physical exposure" OR "physical exertion"):ab,ti,kw

Table 2. Methodological quality scale obtained from Kmet e.a. (2004)

	Criteria	Yes (2)	Partial (1)	No (0)	N/A
1.	Question / objective sufficiently described?				
2.	Study design evident and appropriate?				
3.	Method of subject/comparison group selection *or* source of information/input variables described and appropriate?				
4.	Subject (and comparison group, if applicable) characteristics sufficiently described?				
5.a.	Exposure measure(s) well defined and robust to measurement / misclassification bias? Means of assessment reported?				
5.b.	Outcome measure(s) well defined and robust to measurement / misclassification bias? Means of assessment reported?				
6.	Sample size appropriate?				
7.	Analytic methods described/justified and appropriate?				
8.	Some estimate of variance is reported for the main results?				
9.	Controlled for confounding?				
10.	Results reported in sufficient detail?				
11.	Conclusions supported by the results?				
	Summary score				

The summary score was calculated as: total sum[(number of 'yes' × 2) + (number of 'partial' × 1)]/total possible sum[22 − (number of 'N/A' × 2)], with a maximum possible total score of 1.

Table S3:. Categories of occupational physical activity of studies included in the meta-analysis, as defined during a consensus meeting (with authors BC, PC, MH, AvdB and/or NK).

		Occupational physical activity categories[1]			
	First author; Year	Sedentary	Low	Moderate	High
1	Barengo 2004		Low	Moderate	High
2	Mok, 2019	Sedentary	Standing occupation		Manual occupation
3	Chasland; 2017		Low		High
4	Graff-Iversen 2007	Sedentary	Light	Moderate	Heavy
5	Harari 2015		Not active - light		Moderate - hard
6	Holtermann 2010A	Sedentary		Moderate	High
7	Holtermann 2016	Sedentary		Moderate	High
8	Huerta 2016		Sedentary		Non-sedentary
9	Krause, 2017		Low		High
10	Menotti 2016	Sedentary		Moderate	Vigorous
11	Moe 2013	Sedentary	Walking/lifting		Physical work
12	Rosengren 1997	Sedentary work	Light mobile	Heavy work	Very heavy
13	Smigielski; 2016		Low		High
14	Stamatakis 2013	Sitting		Stand/walk	
15	Yu 2003	Quartile 1	Quartile 2	Quartile 3	Quartile 4
16	Hayashi, 2016	Mostly sitting	Sitting and standing	Mostly standing<br>Standing and walking	
17	Hermansen, 2019	Mostly sitting	Walking	Walking and lifting	Heavy manual work
18	Wanner, 2019[2]		Low	Moderate	High
19	Wanner, 2019[3]		Low	Moderate	High
20	Bennett, 2017	0.1-5.9[4]	6.0-13.8[4]	13.9-25.7[4]	>25.8[4]
21	Mikkola, 2019	Quartile 1	Quartile 2	Quartile 3	Quartile 4
22	Bahls, 2018[5]		Low		High
23	Bahls, 2018[6]		Low		High

[1]According to the PA continuum(1, 2)
[2]NRP1A Study
[3]MONICA
[4]MET-h/day
[5]SHIP-study
[6]CARLA-study
Green color is indicating the reference group.

Table S4: Exclusion list after full text.

		Article	Reason
1	1	Albert, MA, Glynn, RJ, Buring, J, Ridker, PM. Impact of traditional and novel risk factors on the relationship between socioeconomic status and incident cardiovascular events. Circulation 2006,114(24):2619-2626	Study is not about occupational PA
2	2	Armstrong, DL, Castorina, J. Community occupational structure, basic services, and coronary mortality in Washington state, 1980-1994. Ann Epidemiol 1998,8(6):370-377	Study is not about occupational PA
3	3	Arndt, V., Rothenbacher, D., Zschenderlein, B., Schuberth, S., Brenner, H. Body mass index and premature mortality in physically heavily working men--a ten-year follow-up of 20,000 construction workers. J Occup Environ Med 2007,49(8):913-21	Study is not about occupational PA
4	4	Bjorck, L, Novak, M, Schaufelberger, M, Giang, KW, Rosengren, A. Body weight in midlife and long-term risk of developing heart failure-a 35-year follow-up of the primary prevention study in Gothenburg, Sweden. BMC Cardiovasc Disord 2015,15:19	Study is not about occupational PA
5	5	Blair, A, Sandler, DP, Tarone, R, Lubin, J, Thomas, K, Hoppin, JA, Samanic, C, Coble, J, Kamel, F, Knott, C, Dosemeci, M, Zahm, SH, Lynch, CF, Rothman, N, Alavanja, MC. Mortality among participants in the agricultural health study. Ann Epidemiol Apr 2005,15(4):279-85	Study is not about occupational PA
6	6	Boice, JD, Mandel, JS, Doody, MM, Yoder, RC, McGowan, R. A health survey of radiologic technologists. Cancer 1992,69(2):586-98	Study is not about occupational PA
7	7	Calle, EE, Murphy, TK, Rodriguez, C, Thun, MJ, Heath, CW. Occupation and breast cancer mortality in a prospective cohort of US women. Am J Epidemiol 15 1998,148(2):191-7	Study is not about occupational PA
8	8	Damlund, M, Goth, S, Hasle, P, Jeune, B, Munk, K. The incidence of disability pensions and mortality among semi-skilled construction workers in Copenhagen. A retrospective cohort study with two control groups. Scand J Soc Med 1982,10(2):43-7	Study is not about occupational PA
9	9	Farioli, A, Yang, J, Teehan, D, Baur, DM, Smith, DL, Kales, SN. Duty-related risk of sudden cardiac death among young US firefighters. Occup Med 2014,64(6):428-35	Study is not about occupational PA
10	10	Gallo, V, Mackenbach, JP, Ezzati, M, Menvielle, G, Kunst, AE, et al. Social inequalities and mortality in Europe--results from a large multi-national cohort. PLoS One 2012,7(7):e39013	Study is not about occupational PA
11	11	Hara, M, Mori, M, Nishizumi, M. Differences in lifestyle-related risk factors for death by occupational groups, a prospective study. J Occup Health 1999,41(3):137-143	Study is not about occupational PA
12	12	Harmsen, P, Rosengren, A, Tsipogianni, A, Wilhelmsen, L. Risk factors for stroke in middle-aged men in Goteborg, Sweden. Stroke 1990,21(2):223-9	Study is not about occupational PA
13	13	Holtermann, A, Mortensen, OS, Burr, H, Sogaard, K, Gyntelberg, F, Suadicani, P. Physical fitness and perceived psychological pressure at work: 30-year ischemic heart disease and all-cause mortality in the Copenhagen Male Study. J Occup Environ Med 2011,53(7):743-50	Study is not about occupational PA
14	14	Holtermann, A, Mortensen, OS, Burr, H, Sogaard, K, Gyntelberg, F, Suadicani, P. Long work hours and physical fitness: 30-year risk of ischaemic heart disease and all-cause mortality among middle-aged Caucasian men. Heart 2010,96(20):1638-44	Study is not about occupational PA
15	15	Khaw, KT, Jakes, R, Bingham, S, Welch, A, Luben, R, Day, N, Wareham, N. Work and leisure time physical activity assessed using a simple, pragmatic, validated questionnaire and incident cardiovascular disease and all-cause mortality in men and women:	Study is not about occupational PA

		The European Prospective Investigation into Cancer in Norfolk prospective population study. Int J Epidemiol 2006,35(4):1034-43	
16	16	Kjeldsen, SE, Mundal, R, Sandvik, L, Erikssen, G, Thaulow, E, Erikssen, J. Supine and exercise systolic blood pressure predict cardiovascular death in middle-aged men. J Hypertens 2001,19(8):1343-8	Study is not about occupational PA
17	17	Moller, SV, Hannerz, H, Hansen, AM, Burr, H, Holtermann, A. Multi-wave cohort study of sedentary work and risk of ischemic heart disease. Scand J Work Environ Health 1 2016,42(1):43-51	Study is not about occupational PA
18	18	Myint, PK, Luben, RN, Wareham, NJ, Welch, AA, Bingham, SA, Day, NE, Khaw, KT. Combined work and leisure physical activity and risk of stroke in men and women in the European prospective investigation into Cancer-Norfolk Prospective Population Study. Neuroepidemiology 2006,27(3):122-9	Study is not about occupational PA
19	19	McFadden, E, Luben, R, Wareham, N, Bingham, S, Khaw, KT. Occupational social class, risk factors and cardiovascular disease incidence in men and women: a prospective study in the European Prospective Investigation of Cancer and Nutrition in Norfolk (EPIC-Norfolk) cohort. Eur J Epidemiol 2008,23(7):449-58	Study is not about occupational PA
20	20	Shirom, A, Toker, S, Jacobson, O, Balicer, RD. Feeling vigorous and the risks of all-cause mortality, ischemic heart disease, and diabetes: a 20-year follow-up of healthy employees. Psychosom Med 2010,72(8):727-33	Study is not about occupational PA
21	21	Sobolski, J, Kornitzer, M, de Backer, G. Protection against ischemic heart disease in the Belgian Physical Fitness Study: Physical fitness rather than physical activity? Am J Epidemiol 1987,125(4):601-610	Study is not about occupational PA
22	22	Singer, S.; Bartels, M.; Briest, S.; Einenkel, J.; Niederwieser, D.; Papsdorf, K.; Stolzenburg, J. U.; Künstler, S.; Taubenheim, S.; Krauß, O. Socio-economic disparities in long-term cancer survival—10 year follow-up with individual patient data Supportive Care in Cancer 2017;25(5):1391-1399	Study is not about occupational PA
23	23	Loprinzi, P. D.; Edwards, M. K.; Sng, E.; Addoh, O. Sedentary behavior and residual-specific mortality Health Promot Perspect 2016;6(4):196-201	Study is not about occupational PA
24	24	Loprinzi, P. D.; Sng, E.; Addoh, O. Physical Activity and Residual-Specific Mortality among Adults in the United States Med Sci Sports Exerc 2016;48(9):1730-6	Study is not about occupational PA
25	25	Loprinzi, P. D.; Loenneke, J. P. Mortality risk and perceived quality of life as a function of waking time in discretionary movement-based behaviors: isotemporal substitution effects Qual Life Res 2017;26(2):343-348	Study is not about occupational PA
26	26	Loprinzi, P. D.; Davis, R. E. Psycho-socioeconomic bio-behavioral associations on all-cause mortality: cohort study Health Promot Perspect 2016;6(2):66-70	Study is not about occupational PA
27	27	Loprinzi, P. D.; Addoh, O.; Joyner, C. Multimorbidity, mortality, and physical activity Chronic Illn 2016;12(4):272-280	Study is not about occupational PA
28	28	Jensen, M. T.; Holtermann, A.; Bay, H.; Gyntelberg, F. Cardiorespiratory fitness and death from cancer: a 42-year follow-up from the Copenhagen Male Study Br J Sports Med 2017;51(18):1364-1369	Study is not about occupational PA
29	29	Dorn, J. P., Cerny, F. J., Epstein, L. H., Naughton, J., Vena, J. E., Winkelstein, W., Jr., . . . Trevisan, M. (1999). Work and leisure time physical activity and mortality in men and women from a general population sample. *Ann Epidemiol, 9*(6)	Study is not about occupational PA
30	30	Edwards, M. K., Shivappa, N., Mann, J. R., Hebert, J. R., Wirth, M. D., & Loprinzi, P. D. (2018). The association between physical activity	Study is not about occupational PA

		and dietary inflammatory index on mortality risk in U.S. adults. *Phys Sportsmed, 46*(2), 249-254.	
31	31	Kim, H. C., Shin, W. Y., Lee, T., & Jeon, D. H. (2018). Diabetes, frequency of exercise, and mortality over 12 years. *Circulation, 137*.	Study is not about occupational PA
32	32	Koolhaas, C. M., Dhana, K., Schoufour, J. D., Lahousse, L., Rooij, F. J. A. v., Ikram, M. A., . . . van Rooij, F. J. A. (2018). Physical activity and cause-specific mortality: the Rotterdam Study. *Int J Epidemiol, 47*(5), 1705-1713.	Study is not about occupational PA
33	33	Stringhini, S., Zaninotto, P., Kumari, M., Kivimaki, M., Lassale, C., & Batty, G. D. (2018). Socio-economic trajectories and cardiovascular disease mortality in older people: the English Longitudinal Study of Ageing. *Int J Epidemiol, 47*(1)	Study is not about occupational PA
34	34	van Hedel, K., van Lenthe, F. J., Oude Groeniger, J., & Mackenbach, J. P. (2018). What's the difference? A gender perspective on understanding educational inequalities in all-cause and cause-specific mortality. *BMC Public Health, 18*(1)	Study is not about occupational PA
35	35	Kapral, M. K., Austin, P. C., Jeyakumar, G., Hall, R., Chu, A., Khan, A. M., . . . Tu, J. V. (2019). Rural-urban differences in stroke risk factors, incidence, and mortality in people with and without prior stroke: The CANHEART stroke study. *Circulation: Cardiovascular Quality and Outcomes, 12*(2).	Study is not about occupational PA
36	36	Yerramalla, M. S., Fayosse, A., Dugravot, A., Tabak, A. G., Kivimäki, M., Singh-Manoux, A., & Sabia, S. (2020). Association of moderate and vigorous physical activity with incidence of type 2 diabetes and subsequent mortality: 27 year follow-up of the Whitehall II study. *Diabetologia, 63*(3), 537-548.	Study is not about occupational PA
37	1	Crespo, CJ, Garcia-Palmieri, MR, Smit, E, Lee, IM, McGee, D, Muti, P, Figueroa Valle, NR, Ramierez-Marrero, FA, Freudenheim, JL, Sorlie, P. Physical activity and prostate cancer mortality in Puerto Rican men. J Phys Act Health 2008,5(6):918-29	Occupational and leisure-time PA in one variable
38	2	Hinkle, LE, Thaler, HT, Merke, DP, Renier-Berg, D, Morton, NE. The risk factors for arrhythmic death in a sample of men followed for 20 years. Am J Epidemiol 1988,127(3):500-15	Occupational and leisure-time PA in one variable
39	3	Inoue, M, Iso, H, Yamamoto, S, Kurahashi, N, Iwasaki, M, Sasazuki, S, Tsugane, S. Daily total physical activity level and premature death in men and women: results from a large-scale population-based cohort study in Japan (JPHC study). Ann Epidemiol 2008,18(7):522-30	Occupational and leisure-time PA in one variable
40	4	Kannel, WB, Sorlie, P. Some health benefits of physical activity. The Framingham Study. Arch Intern Med 1979,139(8):857-61	Occupational and leisure-time PA in one variable
41	5	Matthews CE, Moore SC, Sampson J, Blair A, Xiao Q, Keadle SK, Hollenbeck A., Park Y. Mortality Benefits for Replacing Sitting Time with Different Physical Activities. Med Sci Sports Exerc 2015,47(9):1833-1839	Occupational and leisure-time PA in one variable
42	6	Orsini, N, Bellocco, R, Bottai, M, Pagano, M, Michaelsson, K, Wolk, A. Combined effects of obesity and physical activity in predicting mortality among men. J Intern Med 2008,264(5):442-451	Occupational and leisure-time PA in one variable
43	7	Padyab, M, Blomstedt, Y, Norberg, M. No association found between cardiovascular mortality, and job demands and decision latitude: experience from the Vasterbotten Intervention Programme in Sweden. Soc Sci Med 2014,117:58-66	Occupational and leisure-time PA in one variable
44	8	Pulsford, RM, Stamatakis, E, Britton, AR, Brunner, EJ, Hillsdon, M. Associations of sitting behaviours with all-cause mortality over a 16-year follow-up: the Whitehall II study. Int J Epidemiol 2015,44(6):1909-16	Occupational and leisure-time PA in one variable
45	9	Siscovick, DS, Ekelund, LG, Hyde, JS, Johnson, JL, Gordon, DJ, LaRosa, JC. Physical activity and coronary heart disease among asymptomatic	Occupational and leisure-time PA in one variable

		hypercholesterolemic men (the Lipid Research Clinics Coronary Primary Prevention Trial). Am J Public Health 1988,78(11):1428-31	
46	10	Lear, S.; Gasevic, D.; Hu, W.; Rangaran, S.; Leong, D. P.; Teo, K. K.; Yusuf, S. The effect of overall and types of physical activity on mortality and cardiovascular events in 17 countries: Results from the prospective urban rural epidemiologic (Pure) study Global Heart 2016;11(2):e1 2016	Occupational and leisure-time PA in one variable
47	11	Jayasekara, H.; English, D. R.; Haydon, A.; Hodge, A. M.; Lynch, B. M.; Rosty, C.; Williamson, E. J.; Clendenning, M.; Southey, M. C.; Jenkins, M. A.; Room, R.; Hopper, J. L.; Milne, R. L.; Buchanan, D. D.; Giles, G. G.; MacInnis, R. J. Associations of alcohol intake, smoking, physical activity and obesity with survival following colorectal cancer diagnosis by stage, anatomic site and tumor molecular subtype Int J Cancer 2017	Occupational and leisure-time PA in one variable
48	12	Evenson, K. R.; Wen, F.; Herring, A. H. Associations of Accelerometry-Assessed and Self-Reported Physical Activity and Sedentary Behavior with All-Cause and Cardiovascular Mortality among US Adults American Journal of Epidemiology 2016;184(9):621-632	Occupational and leisure-time PA in one variable
49	13	Edwards, M. K.; Loprinzi, P. D. All-cause mortality risk as a function of sedentary behavior, moderate-to-vigorous physical activity and cardiorespiratory fitness Phys Sportsmed 2016;44(3):223-30	Occupational and leisure-time PA in one variable
50	14	Byrne, D. W.; Rolando, L. A.; Aliyu, M. H.; McGown, P. W.; Connor, L. R.; Awalt, B. M.; Holmes, M. C.; Wang, L.; Yarbrough, M. I. Modifiable Healthy Lifestyle Behaviors: 10-Year Health Outcomes From a Health Promotion Program American Journal of Preventive Medicine 2016;51(6):1027-1037	Occupational and leisure-time PA in one variable
51	15	Berstad, P.; Botteri, E.; Larsen, I. K.; Loberg, M.; Kalager, M.; Holme, O.; Bretthauer, M.; Hoff, G. Lifestyle changes at middle age and mortality: a population-based prospective cohort study J Epidemiol Community Health 2017;71(1):59-66	Occupational and leisure-time PA in one variable
52	16	Warren Andersen, S.; Zheng, W.; Sonderman, J.; Shu, X. O.; Matthews, C. E.; Yu, D.; Steinwandel, M.; McLaughlin, J. K.; Hargreaves, M. K.; Blot, W. J. Combined Impact of Health Behaviors on Mortality in Low-Income Americans American Journal of Preventive Medicine 2016;51(3):344-355	Occupational and leisure-time PA in one variable
53	17	Bouisset, F., Ruidavets, J.-B., Bongard, V., Taraszkiewicz, D., Bérard, E., Galinier, M., . . . Ferrières, J. (2020). Long-term Prognostic Impact of Physical Activity in Patients With Stable Coronary Heart Disease. *Am J Cardiol, 125*(2), 176-181.	Occupational and leisure-time PA in one variable
54	1	Kittel, F, De Smet, P, Leynen, F, Dramaix, M, De Backer, G, Kornitzer, M. Socio-professional level and long-term mortality in three Belgian large-scale studies. Arch Public Health 2003,61(1-2):3-14	Occupational PA not correctly measured
55	2	Marmot, MG, Smith, GD, Stansfeld, S, Patel, C, North, F, Head, J, White, I, Brunner, E, Feeney, A. Health inequalities among British civil servants: the Whitehall II study. Lancet 8 1991,337(8754):1387-93	Occupational PA not correctly measured
56	3	Pocock, SJ, Shaper, AG, Cook, DG, Phillips, AN, Walker, M. Social class differences in ischaemic heart disease in British men. Lancet 25 1987,2(8552):197-201	Occupational PA not correctly measured
57	4	Emberson JR, Whincup PH, Morris RW, Walker M. Social class differences in coronary heart disease in middle-aged British men: implications for prevention. Int J Epidemiol 2004;33(2):289-96	Occupational PA not correctly measured
58	5	Ferrario MM, Veronesi G, Chambless LE, Sega R, The contribution of major risk factors and job strain to occupational class differences in coronary heart disease incidence: The MONICA Brianza and PAMELA population-based cohorts. Occup Environ Med 2011;68(10):717-22	Occupational PA not correctly measured

59	6	Johnson, J. V.; Stewart, W.; Hall, E. M.; Fredlund, P.; Theorell, T. Long-term psychosocial work environment and cardiovascular mortality among Swedish men Am J Public Health 1996;86(3):324-31	Occupational PA not correctly measured
60	7	Virtanen SV.; Notkola V. Socioeconomic inequalities in cardiovascular mortality and the role of work: a register study of Finnish men. International journal of Epidemiology 2002;31(3):614-21	Occupational PA not correctly measured
61	8	Ervasti, J., Pietilainen, O., Rahkonen, O., Lahelma, E., Kouvonen, A., Lallukka, T., & Manty, M. (2019). Long-term exposure to heavy physical work, disability pension due to musculoskeletal disorders and all-cause mortality: 20-year follow-up-introducing Helsinki Health Study job exposure matrix. *Int Arch Occup Environ Health, 92*(3), 337-345.	Occupational PA not correctly measured
62	9	Li Y, Sato Y, and Yamaguchi N. Lifestyle factors as predictors of general cardiovascular disease: use for early self-screening. 2014 Jul 26(4):414-24.	Occupational PA not correctly measured
63	1	Kikuchi, H, Inoue, S, Odagiri, Y, Inoue, M, Sawada, N, Tsugane, S. Occupational sitting time and risk of all-cause mortality among Japanese workers. Scand J Work Environ Health 2015,41(6):519-28	Occupational PA was only expressed in sedentary work (yes/no)
64	2	Kim, Y, Wilkens, LR, Park, SY, Goodman, MT, Monroe, KR, Kolonel, LN. Association between various sedentary behaviours and all-cause, cardiovascular disease and cancer mortality: the Multiethnic Cohort Study. Int J Epidemiol 2013,42(4):1040-56	Occupational PA was only expressed in sedentary work (yes/no)
65	3	Hayashi, R.; Iso, H.; Cui, R.; Tamakoshi, A. Occupational physical activity in relation to risk of cardiovascular mortality: The Japan Collaborative Cohort Study for Evaluation for Cancer Risk (JACC Study) Prev Med 2016;89():286-91	Occupational PA was only expressed in sedentary work (yes/no)
66	4	Grunseit, A. C., Chau, J. Y., Rangul, V., Holmen, T. L., & Bauman, A. (2017). Patterns of sitting and mortality in the Nord-Trøndelag health study (HUNT). *International Journal of Behavioral Nutrition and Physical Activity, 14*(1), 8	Occupational PA was only expressed in sedentary work (yes/no)
67	5	Garcia, J. M., Duran, A. T., Schwartz, J. E., Booth, J. N., Hooker, S. P., Willey, J. Z., . . . Diaz, K. M. (2019). Types of sedentary behavior and risk of cardiovascular events and mortality in blacks: The Jackson heart study. *J Am Heart Assoc, 8*(13).	Occupational PA was only expressed in sedentary work (yes/no)
68	1	Hrafnkelsdottir, SM, Torfadottir, JE, Aspelund, T, Magnusson, KT, Tryggvadottir, L, Gudnason, V, Mucci, LA, Stampfer, M, Valdimarsdottir, UA. Physical Activity from Early Adulthood and Risk of Prostate Cancer: A 24-Year Follow-Up Study among Icelandic Men. Cancer Prev Res 2015,8(10):905-11	Not about mortality
69	2	Jensen, G, Nyboe, J, Appleyard, M, Schnohr, P. Risk factors for acute myocardial infarction in Copenhagen, II: Smoking, alcohol intake, physical activity, obesity, oral contraception, diabetes, lipids, and blood pressure. Eur Heart J 1991,12(3):298-308	Not about mortality
70	3	Menotti, A, Lanti, M, Seccareccia, F, Giampaoli, S, Dima, F. Multivariate prediction of the first major cerebrovascular event in an Italian population sample of middle-aged men followed up for 25 years. Stroke 1993,24(1):42-8	Not about mortality
71	4	Rahman, I, Bellavia, A, Wolk, A. Relationship between physical activity and heart failure risk in women. Circulation: Heart Failure 2014,7(6):877-881	Not about mortality
72	5	Sjol, A, Thomsen, KK, Schroll, M, Andersen, LB. Secular trends in acute myocardial infarction in relation to physical activity in the general Danish population. Scand J Med Sci Sports 2003,13(4):224-30	Not about mortality

73	6	Skielboe, A. K.; Marott, J. L.; Dixen, U.; Friberg, J. B.; Jensen, G. B. Occupational physical activity, but not leisure-time physical activity increases the risk of atrial fibrillation: The Copenhagen City Heart Study Eur J Prev Cardiol 2016;23(17):1883-1893	Not about mortality
74	7	Clays E, De Bacquer D, Janssens H, et al. The association between leisure time physical activity and coronary heart disease among men with different physical work demands: a prospective cohort study. Eur J Epidemiol 2013;28(3):241-47	Not about mortality
75	8	Clays, E.; Casini, A.; Van Herck, K.; De Bacquer, D.; Kittel, F.; De Backer, G.; Holtermann, A. Do psychosocial job resources buffer the relation between physical work demands and coronary heart disease? A prospective study among men Int Arch Occup Environ Health 2016;89(8):1299-1307	Not about mortality
76	9	Johnsen, A. M.; Alfredsson, L.; Knutsson, A.; Westerholm, P. J.; Fransson, E. I. Association between occupational physical activity and myocardial infarction: a prospective cohort study BMJ Open 03 2016;6(10):e012692	Not about mortality
77	10	Allesoe, K., Sogaard, K., Aadahl, M., Boyle, E., & Holtermann, A. (2016). Are hypertensive women at additional risk of ischaemic heart disease from physically demanding work? *Eur J Prev Cardiol, 23*(10)	Not about mortality
78	11	Skielboe, A. K., Marott, J. L., Dixen, U., Friberg, J. B., & Jensen, G. B. (2016). Occupational physical activity, but not leisure-time physical activity increases the risk of atrial fibrillation: The Copenhagen City Heart Study. *Eur J Prev Cardiol, 23*(17)	Not about mortality
79	12	Wang, C., Roos, A. J. D., Fujishiro, K., Allison, M. A., Wallace, R., Seguin, R. A., . . . De Roos, A. J. (2019). Occupational Physical Activity and Coronary Heart Disease in Women's Health Initiative Observational Study. *Journals of Gerontology Series A: Biological Sciences & Medical Sciences, 74*(12), 1952-1958.	Not about mortality
80	13	Barlas, G., Luben, R. L., Neal, S. R., Wareham, N. J., Khaw, K. T., & Myint, P. K. (2020). Self-Reported Fatigue Predicts Incident Stroke in a General Population: EPIC-Norfolk Prospective Population-Based Study. *Stroke, 51*(4), 1077-1084.	Not about mortality
81	1	Andersen LB, Schnohr P, Schroll M, Hein HO. All-cause mortality associated with physical activity during leisure time, work, sports, and cycling to work. Arch Intern Med 2000;160(11):1621-28	Not about CVD mortality
82	2	Chau JY, Grunseit A, Midthjell K, et al. Sedentary behaviour and risk of mortality from all-causes and cardiometabolic diseases in adults: evidence from the HUNT3 population cohort. Br J Sports Med 2015;49(11):737-42	Not about CVD mortality
83	3	Etemadi A, Abnet CC, Kamangar F, et al. Impact of body size and physical activity during adolescence and adult life on overall and cause-specific mortality in a large cohort study from Iran. Eur J Epidemiol 2014;29(2):95-109	Not about CVD mortality
84	4	Franzon K, Zethelius B, Cederholm T, Kilander L. Modifiable midlife risk factors, independent aging, and survival in older men: report on long-term follow-up of the Uppsala Longitudinal Study of Adult Men cohort. J Am Geriatr Soc 2015;63(5):877-85	Not about CVD mortality
85	5	Hemmingsson T, Lundberg I. Can large relative mortality differences between socio-economic groups among Swedish men be explained by risk indicator-associated social mobility? Eur J Public Health 2005;15(5):518-22	Not about CVD mortality
86	6	Holtermann A, Burr H, Hansen JV, Krause N, Sogaard K, Mortensen OS. Occupational physical activity and mortality among Danish workers. Int Arch Occup Environ Health 2012;85(3):305-10	Not about CVD mortality

87	7	Holtermann A, Marott JL, Gyntelberg F, et al. Occupational and leisure time physical activity: risk of all-cause mortality and myocardial infarction in the Copenhagen City Heart Study. A prospective cohort study. BMJ Open 2012;2(1):e000556	Not about CVD mortality
88	8	Hu GC, Chien KL, Hsieh SF, Chen CY, Tsai WH, Su TC. Occupational versus leisure-time physical activity in reducing cardiovascular risks and mortality among ethnic Chinese adults in Taiwan. Asia Pac J Public Health 2014;26(6):604-13	Not about CVD mortality
89	9	Lapidus L, Bengtsson C. Socioeconomic factors and physical activity in relation to cardiovascular disease and death. A 12 year follow up of participants in a population study of women in Gothenburg, Sweden. Br Heart J 1986;55(3):295-301	Not about CVD mortality
90	10	Menotti A, Lanti M, Maiani G, Kromhout D. Determinants of longevity and all-cause mortality among middle-aged men. Role of 48 personal characteristics in a 40-year follow-up of Italian Rural Areas in the Seven Countries Study. Aging Clin Exp Res 2006;18(5):394-406	Not about CVD mortality
91	11	Norman, A., Moradi, T., Gridley, G., Dosemeci, M., Rydh, B., Nyren, O., Wolk, A. Occupational physical activity and risk for prostate cancer in a nationwide cohort study in Sweden. Br J Cancer 2002,86(1):70-5	Not about CVD mortality
92	12	Orsini, N., Bellocco, R., Bottai, M., Pagano, M., Andersson, S. O., Johansson, J. E., Giovannucci, E., Wolk, A. A prospective study of lifetime physical activity and prostate cancer incidence and mortality. Br J Cancer 2009,101(11):1932-8	Not about CVD mortality
93	13	Petersen CB, Eriksen L, Tolstrup JS, Sogaard K, Gronbaek M, Holtermann A. Occupational heavy lifting and risk of ischemic heart disease and all-cause mortality. BMC Public Health 2012;12:1070	Not about CVD mortality
94	14	Richard A, Martin B, Wanner M, Eichholzer M, Rohrmann S. Effects of leisure-time and occupational physical activity on total mortality risk in NHANES III according to sex, ethnicity, central obesity, and age. J Phys Act Health 2015;12(2):184-92	Not about CVD mortality
95	15	Stender M, Hense HW, Doring A, Keil U. Physical activity at work and cardiovascular disease risk: results from the MONICA Augsburg study. Int J Epidemiol 1993;22(4):644-50	Not about CVD mortality
96	16	Wanner M, Tarnutzer S, Martin BW, et al. Impact of different domains of physical activity on cause-specific mortality: a longitudinal study. Prev Med 2014;62:89-95	Not about CVD mortality
97	17	Turi, B. C.; Codogno, J. S.; Fernandes, R. A.; Sui, X.; Lavie, C. J.; Blair, S. N.; Monteiro, H. L. Association of Different Physical Activity Domains on All-Cause Mortality in Adults Participating in Primary Care in the Brazilian National Health System: 4-Year Follow-up J Phys Act Health 2017;14(1):45-51	Not about CVD mortality
98	18	Clays, E.; Lidegaard, M.; De Bacquer, D.; Van Herck, K.; De Backer, G.; Kittel, F.; de Smet, P.; Holtermann, A. The combined relationship of occupational and leisure-time physical activity with all-cause mortality among men, accounting for physical fitness Am J Epidemiol 2014;179(5):559-661	Not about CVD mortality
99	19	Robsahm, T. E.; Falk, R. S.; Heir, T.; Sandvik, L.; Vos, L.; Erikssen, J. E.; Tretli, S. Measured cardiorespiratory fitness and self-reported physical activity: associations with cancer risk and death in a long-term prospective cohort study Cancer Med 2016;5(8):2136-44	Not about CVD mortality
100	20	Sydén, L., & berg, J. (2017). The contribution of alcohol use and other lifestyle factors to socioeconomic differences in all-cause mortality in a Swedish cohort. *Drug Alcohol Rev, 36*(5)	Not about CVD mortality

101	1	Eaton CB, Medalie JH, Flocke SA, Zyzanski SJ, Yaari S, Goldbourt U. Self-reported physical activity predicts long-term coronary heart disease and all-cause mortalities. Twenty-one-year follow-up of the Israeli Ischemic Heart Disease Study. Arch Fam Med 1995;4(323-329)	Not properly adjusted for relevant factors
102	2	Holme I, Helgeland A, Hjermann I, Leren P, Lund-Larsen PG. Physical activity at work and at leisure in relation to coronary risk factors and social class. A 4-year mortality follow-up. The Oslo study. Acta Med Scand 1981;4(277-283)	Not properly adjusted for relevant factors
103	3	Johansson S, Rosengren A, Tsipogianni A, Ulvenstam G, Wiklund I, Wilhelmsen L. Physical inactivity as a risk factor for primary and secondary coronary events in Goteborg, Sweden. Eur Heart J 1988;9(Suppl L):8-19	Not properly adjusted for relevant factors
104	4	Kannel WB, Belanger A, D'Agostino R, Israel I. Physical activity and physical demand on the job and risk of cardiovascular disease and death: the Framingham Study. Am Heart J 1986;112(4):820-25	Not properly adjusted for relevant factors
105	5	Lissner L, Bengtsson C, Bjorkelund C, Wedel H. Physical activity levels and changes in relation to longevity. A prospective study of Swedish women. Am J Epidemiol 1996;143(1):54-62	Not properly adjusted for relevant factors
106	6	Menotti A, Lanti M, Maiani G, Kromhout D. Forty-year mortality from cardiovascular diseases and their risk factors in men of the Italian rural areas of the Seven Countries Study. Acta Cardiol 2005;60(5):521-31	Not properly adjusted for relevant factors
107	7	Menotti A, Puddu PE, Lanti M, Maiani G, Catasta G, Fidanza AA. Lifestyle habits and mortality from all and specific causes of death: 40-year follow-up in the Italian Rural Areas of the Seven Countries Study. J Nutr Health Aging 2014;18(3):314-21	Not properly adjusted for relevant factors
108	8	Menotti A, Puddu V. Death rates among the Italian railroad employees, with special reference to coronary heart disease and physical activity at work. Environ Res 1976;11(3):331-42	Not properly adjusted for relevant factors
109	9	Menotti A, Puddu V. Ten-year mortality from coronary heart disease among 172,000 men classified by occupational physical activity. Scand J Work Environ Health 1979;5(2):100-08	Not properly adjusted for relevant factors
110	10	Menotti A, Seccareccia F. Physical activity at work and job responsibility as risk factors for fatal coronary heart disease and other causes of death. J Epidemiol Community health 1985;39(4):325-29	Not properly adjusted for relevant factors
111	11	Morris JN, Heady JA, Raffle PA, Robers CG, Parks JW. Coronary heart-disease and physical activity of work. Lancet 1953;265(6796):1111-20	Not properly adjusted for relevant factors
112	12	Ostlin P. Occupational history, self-reported chronic illness, and mortality: a follow up of 25,586 Swedish men and women. J Epidemiol Community Health 1990;44(1):12-16	Not properly adjusted for relevant factors
113	13	Paffenbarger RS, Brand RJ, Sholtz RI, Jung DL. Energy expenditure, cigarette smoking, and blood pressure level as related to death from specific diseases. Am J Epidemiol 1978;108(1):12-18	Not properly adjusted for relevant factors
114	14	Paffenbarger RS, Hale WE. Work activity and coronary heart mortality. N Engl J Med 1975;292(11):545-50	Not properly adjusted for relevant factors
115	15	Paffenbarger RS, Hale WE, Brand RJ, Hyde RT. Work-energy level, personal characteristics, and fatal heart attack: a birth-cohort effect. Am J Epidemiol 1977;105(3):200-13	Not properly adjusted for relevant factors
116	16	Paffenbarger RS, Laughlin ME, Gima AS, Black RA. Work activity of longshoremen as related to death from coronary heart disease and stroke. N Engl J Med 1970;282(20):1109-14	Not properly adjusted for relevant factors
117	17	Paffenbarger RS, Gima AS, Laughlin E, Black RA. Characteristics of longshoremen related fatal coronary heart disease and stroke. Am J Public Health 1971;61(7):1362-70	Not properly adjusted for relevant factors

118	18	Brand, R. J.; Paffenbarger Jr, R. S.; Sholtz, R. I.; Kampert, J. B. Work activity and fatal heart attack studied by multiple logistic risk analysis Am J Epidemiol 1979;110(1):52-62	Not properly adjusted for relevant factors
119	19	Autenrieth CS, Baumert J, Baumeister SE, Fischer B, Peters A, Doring A, et al. Association between domains of physical activity and all-cause, cardiovascular and cancer mortality. Eur J Epidemiol. 2011 Feb 26(2):91-99	Not properly adjusted for relevant factors
120	20	Haheim L, Holme I, Hjermann I, Leren P, Risk factors of stroke incidence and mortality; A 12 year Follow-up of the Oslo study.	Not properly adjusted for relevant factors
121	21	Sakaue A, Adachi H, Enomoto M, Fukami A, et al. Association between physical activity, occupational sitting time and mortality in a general population: An 18-year prospective survey in Tanushimaru, Japan. European Journal of Preventive Cardiology 2018	Not properly adjusted for relevant factors
122	1	Holme, I, Solberg, LA, Weissfeld, L, Helgeland, A, Hjermann, I, Leren, P, Strong, JP, Williams, OD. Coronary risk factors and their pathway of action through coronary raised lesions, coronary stenoses and coronary death. Multivariate statistical analysis of an autopsy series: the Oslo Study. Am J Cardiol 1985,55(1):40-7	Although occupational PA and mortality were both measured, the association between the two has not been assessed
123	2	Holtermann, A, Marott, JL, Gyntelberg, F, Sogaard, K, Suadicani, P, Mortensen, OS, Prescott, E, Schnohr, P. Does the benefit on survival from leisure time physical activity depend on physical activity at work? A prospective cohort study. PLoS One 2013,8(1):e54548	Although occupational PA and mortality were both measured, the association between the two has not been assessed
124	3	Holtermann, A, Mortensen, OS, Sogaard, K, Gyntelberg, F, Suadicani, P. Risk factors for ischaemic heart disease mortality among men with different occupational physical demands. A 30-year prospective cohort study. BMJ Open 2012,2(1):e000279	Although occupational PA and mortality were both measured, the association between the two has not been assessed
125	4	Korshoj, M, Lidegaard, M, Kittel, F, Van Herck, K, De Backer, G, De Bacquer, D, Holtermann, A, Clays, E. The relation of ambulatory heart rate with all-cause mortality among middle-aged men: a prospective cohort study PLoS One 2015,10(3):e0121729	Although occupational PA and mortality were both measured, the association between the two has not been assessed
126	5	Hart CL, Smith GD, Blane D. Social mobility and 21 year mortality in a cohort of Scottish men. Soc Sci Med 1998;47(8):1121-30	Although occupational PA and mortality were both measured, the association between the two has not been assessed
127	6	Holtermann A, Mortensen OS, Burr H, Sogaard K, Gyntelberg F, Suadicani P. The interplay between physical activity at work and during leisure time--risk of ischemic heart disease and all-cause mortality in middle-aged Caucasian men. Scand J Work Environ Health 2009;35(8):466-74	Although occupational PA and mortality were both measured, the association between the two has not been assessed
128	1	Bahls, M, Baumeister, S, Völzke, H, Gläser, S, Leitzmann, M, Felix, SB, Dörr, M. Voluntary and occupational physical activity have different effects on mortality. Circulation 2015,132	Conference abstract
129	2	Blair, S. Physical inactivity: A major public health problem. Obesity Reviews 2014,15:5	Conference abstract
130	3	Blair, S. Physical activity: Impact on mortality and morbidity. Ann Nutr Metab 2013,63:27	Conference abstract
131	4	Dorn, JM, Hovey, K, Trevisan, M. Meeting 2008 physical activity guidelines for americans reduces risk of recurrent CVD events in MI survivors: The western new york acute MI study (1996-2006). Circulation 2012,125(10):	Conference abstract

132	5	Friedenreich, C, Kopciuk, K, Wang, Q, McGregor, S, Angyalfi, S, Courneya, K. Pre- and post-diagnosis physical activity and survival after prostate cancer. J Sci Med *Sport* 2012,15:S334-S335	Conference abstract
133	6	Goldbourt, U. Physical activity on and off the job: Conflicting associations with long-termstrokemortality. Am J Epidemiol 2011,173:S96	Conference abstract
134	7	Golubic, R, Ekelund, U, Luben, R, Khaw, K, Wareham, N, Brage, S. Does total physical activity modify the association between working hours and all-cause mortality? The EPIC-Norfolk cohort. J Sci Med *Sport* 2012,15:S28	Conference abstract
135	8	Holtermann, A, Mortensen, OS, Burr, H, Søgaard, K, Gyntelberg, F, Suadicani, P. Physical work demands and physical fitness in low social classes. Occup Environ Med 2011,68:A53-A54	Conference abstract
136	9	Kokkinos, P, Faselis, C, Myers, J, Manolis, T, Pittaras, A, Kyritsi, F, Doumas, M, Papademetriou, V. Mortality risk and exercise capacity associations in hypertensives according to BMI levels. J Hypertens 2010,28:e229	Conference abstract
137	10	Parekh, N, Lin, Y, Vadiveloo, M, Fitzgerald, N, Lu-Yao, G. Longitudinal associations of physical activity and cancer mortality - The third national health and nutrition examination survey. FASEB Journal 2010,24:	Conference abstract
138	11	Punsar, S., Karvonen, M. J. Physical activity and coronary heart disease in populations from East and West Finland. Advances in Cardiology 1976, 18:196-207	Conference abstract
139	12	Singh, P.; Almarzooq, Z.; Roman, M.; Devereux, R. Gender differences in physical activity levels on cardiovascular events in diabetes: The strong heart study Circulation 2016;133(): 2016	Conference abstract
141	13	Singh, P.; Almarzooq, Z.; Roman, M.; Devereux, R. Physical activity decreases cardiovascular events via an inflammatory pathway: The strong heart study Circulation 2016;133(): 2016	Conference abstract
141	14	Krause, N.; Arah, O.; Kauhanen, J. Occupational and leisure time physical activity, fitness, coronary heart disease, and 22-year mortality: Results from the Kuopio ischemic heart disease risk factor study Eur J Prev Cardiol 2017;24(2):11-12 2017	Conference abstract
142	15	Tatishvili, S.; Esquirol, Y.; Ruidavets, J. B.; Ferrieres, J. Does leisure physical activity efficiently decrease the consequences of occupational social inequalities on cardiovascular diseases? Prime study Archives of Cardiovascular Diseases Supplements 2016;8(1):97-98 2016	Conference abstract
143	16	Bauman, A., Ding, D., Pont, S., Berger, C., Adachi, J. D., Hopman, W. M., . . . Goltzman, D. (2015). Sedentary behaviour, sitting and mortality in the canadian multicentre osteoporosis study (CaMOS)-cross-sectional and 10-year prospective data. *Journal of Bone and Mineral Research, 30.*	Conference abstract
144	17	Bennett, D., Li, L., Du, H., Gou, Y., Bian, Z., Chen, J., & Chen, Z. (2016). Cardiovascular benefits of total and domain specific physical activity in resource poor settings: Findings from a 7-year prospective study of 0.5 million chinese adults. *Circulation, 134*.	Conference abstract
145	18	Ferrières, J., Taraszkiewicz, D., Bongard, V., Berard, E., Kai, S. H. Y., & Ruidavets, J. B. (2019). Long-term impact of physical activity on mortality in stable coronary heart disease: The GENES study. *Archives of Cardiovascular Diseases Supplements, 11*(1), 10.	Conference abstract
146	1	Chen, LJ, Fox, KR, Ku, PW, Sun, WJ, Chou, P. Prospective associations between household-, work-, and leisure-based physical activity and all-cause mortality among older Taiwanese adults. Asia Pac J Public Health 2012,24(5):795-805	Non adult (18-65) population

147	2	Glass, TA, Mendes De Leon, C, Marottoli, RA, Berkman, LF. Population based study of social and productive activities as predictors of survival among elderly Americans. BMJ 1999,319(7208):478-483	Non adult (18-65) population
148	3	Tobiasz-Adamczyk, B, Brzyski, P, Florek, M, Brzyska, M. Job stress and mortality in older age. Int J Occup Med Environ Health 2013,26(3):349-62	Non adult (18-65) population
149	1	Friedenreich, CM, Gregory, J, Kopciuk, KA, Mackey, JR, Courneya, KS. Prospective cohort study of lifetime physical activity and breast cancer survival. Int J Cancer 2009,124(8):1954-62	Clinical Population
150	2	Holtermann, A, Mortensen, OS, Burr, H, Sogaard, K, Gyntelberg, F, Suadicani, P. Fitness, work, and leisure-time physical activity and ischaemic heart disease and all-cause mortality among men with pre-existing cardiovascular disease. Scand J Work Environ Health 2010,36(5):366-72	Clinical Population
151	3	Hu, G, Eriksson, J, Barengo, NC, Lakka, TA, Valle, TT, Nissinen, A, Jousilahti, P, Tuomilehto, J. Occupational, commuting, and leisure-time physical activity in relation to total and cardiovascular mortality among Finnish subjects with type 2 diabetes. Circulation 2004,110(6):666-73	Clinical Population
152	4	Hu, G, Jousilahti, P, Antikainen, R, Tuomilehto, J. Occupational, commuting, and leisure-time physical activity in relation to cardiovascular mortality among finnish subjects with hypertension. Am J Hypertens 2007,20(12):1242-50	Clinical Population
153	5	Iijima, K, Iimuro, S, Shinozaki, T, Ohashi, Y, Sakurai, T, Umegaki, H, Araki, A, Ouchi, Y, Ito, H. Lower physical activity is a strong predictor of cardiovascular events in elderly patients with type 2 diabetes mellitus beyond traditional risk factors: the Japanese Elderly Diabetes Intervention Trial. Geriatr Gerontol Int 2012,12 Suppl 1:77-87	Clinical Population
154	6	Loprinzi, PD. The effects of objectively-measured, free-living daily ambulatory movement on mortality in a national sample of adults with diabetes. Physiol Behav 1 2016,154:126-8	Clinical Population
155	7	Loprinzi, PD. Accelerometer-determined physical activity and all-cause mortality in a national prospective cohort study of hypertensive adults. J Hypertens 29 2016,:	Clinical Population
156	8	Loprinzi, PD., Joyner, C. Accelerometer-determined physical activity and mortality in a national prospective cohort study: Considerations by visual acuity. Prev Med 6 2016,87:18-21	Clinical Population
157	9	Loprinzi, PD, Walker, JF. Increased daily movement associates with reduced mortality among COPD patients having systemic inflammation. Int J Clin Pract 2016,70(3):286-91	Clinical Population
158	10	Pinelli, L, Marini, A, Voronovitsky, G, Lugo, M. Habitual physical activity and survival of patients in hemodialysis. NDT Plus 2010,3:iii86	Clinical Population
159	11	Sone, H, Tanaka, S, Tanaka, S, Suzuki, S, Seino, H, Hanyu, O, Sato, A, Toyonaga, T, Okita, K, Ishibashi, S, Kodama, S, Akanuma, Y, Yamada, N. Leisure-time physical activity is a significant predictor of stroke and total mortality in Japanese patients with type 2 diabetes: analysis from the Japan Diabetes Complications Study (JDCS). Diabetologia 2013,56(5):1021-30	Clinical Population
160	12	Sternfeld, B., Weltzien, E., Quesenberry, C. P., Jr., Castillo, A. L., Kwan, M., Slattery, M. L., Caan, B. J. Physical activity and risk of recurrence and mortality in breast cancer survivors: findings from the LACE study. Cancer Epidemiol Biomarkers Prev 2009,18(1):87-95	Clinical Population

161	13	Zelle, DM, Corpeleijn, E, Stolk, RP, de Greef, MH, Gans, RO, van der Heide, JJ, Navis, G, Bakker, SJ. Low physical activity and risk of cardiovascular and all-cause mortality in renal transplant recipients. Clin J Am Soc Nephrol 2011,6(4):898-905	Clinical Population
162	1	Hennekens, CH, Rosner, B, Jesse, MJ, Drolette, ME, Speizer, FE. A retrospective study of physical activity and coronary deaths. Int J Epidemiol 1977,6(3):243-6	Retrospective study
163	1	Hu, GC, Chien, KL, Hsieh, SF, Chen, CY, Tsai, WH, Su, TC. Occupational versus leisure-time physical activity in reducing cardiovascular risks and mortality among ethnic Chinese adults in Taiwan. Asia Pac J Public Health 2014,26(6):604-613	Duplicate
164	2	Marti, B, Minder, CE. [Physical occupational activity and colonic carcinoma mortality in Swiss men 1979-1982]. Soz Praventiv Med 1989,34(1):30-7	Duplicate
165	1	Marti, B, Minder, CE. Occupational physical activity and colon cancer mortality of Swiss men 1979-1982. Soz Praventiv Med 1989,34(1):30-37	Not in English
166	1	Menotti, A, Lanti, M. Coronary risk factors predicting early and distant coronary deaths. CVD Prevention 2000,3(3):205-212	Full text article could not be found

Table S5: Included reviews

Article	Author		Cohort/Study Name	Follow up period [median]	Included/Excluded
1	Barengo, 2004	Finland	FINMONICA/North Karelia Project	20 years	Included
2	Mikkola, 2019	Finland	Helsinki Birth Cohort Study	26 years	Included
3	Krause, 2017	Finland	Kuopio Ischemic Heart Disease (KIDH) Risk factor study	21.7 years	Included
4	Salonen, 1982	Finland	North Karelia project	7 years	Excluded, partially same sample as Barengo.
5	Salonen 1988	Finland	North Karelia project	6 years	Excluded, partially same sample as Barengo
6	Mok, 2019[1]	UK	European Prospective Investigation into Cancer (EPIC) Study	12 years	Included
7	Besson, 2008	UK	European Prospective Investigation into Cancer (EPIC) Study	7 years	Excluded, same sample as Mok
8	Stamatakis, 2013	UK	Health Survey for England and Scottish health survey	12.9 year	Included
9	Yu, 2003	UK	Caerphilly collaborative hearth disease study	10.5 years	Included
10	Chasland, 2017	Australia	Busselton Health Survey	20 years	Included
11	Graff-Iversen, 2007	Norway	The cardiovascular disease study in Norwegian countries	24 years	Included
12	Hermansen, 2019	Norway	The Finnmark study	23.3 years	Included
13	Moe, 2013	Norway	Nord-Trondelag Health Study 2 (HUNT2)	12.4 years	Included
14	Harari, 2015	Israel	The CORDIS Study	22 years	Included
15	Kristah-Boneh 2000	Israel	CORDIS Study	8 years	Excluded, same sample as Harari
16	Holtermann 2010 A	Denmark	Copenhagen Male Study	30 years	Included
17	Holtermann 2010 B	Denmark	Copenhagen Male Study	30 years	Excluded, same population as Holtermann 2010A
18	Holtermann, 2011	Denmark	Copenhagen Male Study	30 years	Excluded, same population as Holtermann 2010A
19	Suadicini, 2001	Denmark	Copenhagen Male Study	8-15-22 years	Excluded, same sample as Holtermann 2010A
20	Holtermann, 2016[3]	Denmark	Copenhagen City Heart Study	18,5 years	Included
21	Huerta, 2016	Spain	Spanish Branch of EPIC / Spain	13.6 years	Included
22	Menotti, 2016	Italy	The Italian Rural Areas of the Seven Countries study of Cardiovascular Diseases	50 years	Included
23	Menotti 2003	Italy	The Italian Rural Areas of the Seven Countries study of Cardiovascular Diseases	35 years	Excluded, same sample as Menotti 2016
24	Italian research group 1982	Italy	The Italian Rural Areas of the Seven Countries study of Cardiovascular Diseases	20 years	Excluded, same sample as Menotti 2016

25	Seccareccia. 1992	Italy	The Italian Rural Areas of the Seven Countries study of Cardiovascular Diseases	25 years	Excluded, same sample as Menotti 2016
26	Rosengren, 1997	Sweden	Multifactor Primary Prevention Study Gotenborg	20 years	Included
27	Smigielski, 2016	Poland	National Multicentre Health Survey (WOBASZ)	3.3 years	Included
28	Hayashi, 2016	Japan	JACC Study	19,2 years	Included
29	Wanner, 2019	Switzerland	NRP1A study and the MONICA study	38 years	Included
30	Bennett, 2019[2]	China	China Kadoorie Biobank	7.5 years	Included
31	Bahls, 2018[4]	Germany	Study of Health in Pomerania (SHIP) and Cardiovascular Disease, Living and Ageing in Halle Study (CARLA)	8.2 years (SHIP)<br>11.5 years (CARLA)	Included

1: Mok (2019) provided new analysis on request

2: Bennett (2019) provided new analysis on request

3: Holtermann (2016); provided new analysis on request

4: Bahls (2018); provided new analysis on request

Table S6: Data Extraction of included studies

	First author, Year;	Study (name, design and follow-up period)	Sample description (n, relevant inclusion/exclusion criteria, %Female, Age, Country; Type of workers).	Description of occupational PA (way of assessment, description of categories, year of occupational PA assessment)	Description of mortality (type of mortality, way of assessment, and incidence over the follow-up period, ICD* code used)	Adjustment	Effect estimates (e.g., HR, RR or OR with 95% confidence interval) Note: Superscript numbers refer to the models specified in the 'adjustment' column
1	Barengo; 2004	Study name: Six independent cross-sectional surveys (within the framework of the North Karelia Project and the FINMONICA/Finrisk studies)<br><br>Study design: Prospective cohort study<br><br>Follow-up period: 20 years (median)	n=32,677<br><br>Inclusion/exclusion: participants with a history of IHD, stroke, heart failure or cancer or who were physically inactive because of severe disease or disability were excluded<br><br>%Females: 16,824 (51%)<br><br>Age: 43.4 (8.4) (males) 43.8 (8.5) (males)<br><br>Country: Finland<br><br>Type of workers: General population	A questionnaire was used assessing occupational PA, categorized into:<br>• Low: mostly sedentary work without much walking (n=4,601 males; n=5,770 females)<br>• Moderate: walking quite a lot at work without lifting or carrying heavy objects (n=3,777 males; n=5,895 females)<br>• High: lots of walking and lifting at work, taking the stairs or walking uphill (n=7,475 males; n=5,159	National registers were used to assess mortality from CVD (n=2,439)<br><br>ICD 9: 390-459*<br>ICD 10: I00-I99*	Three models:<br>1. Age and study year;<br>2. Age, study year, BMI, systolic blood pressure, cholesterol, education, and smoking<br>3. Age, study year, BMI, systolic blood pressure, cholesterol, education, smoking, and other two PA.	Low<br>(males)<br>Death=512<br>HR=Reference[1]<br>HR=Reference[2]<br>HR=Reference[3]<br>(females)<br>Death=302<br>HR=Reference[1]<br>HR=Reference[2]<br>HR=Reference[3]<br><br>Moderate<br>(males)<br>Death=291<br>HR=0.65[0.57 0.76][1]<br>HR=0.75[0.65 0.87][2]<br>HR=0.75[0.64 0.87][3]<br>(females)<br>Death=192<br>HR=0.63[0.53

				females)<br><br>Year of exposure assessment: 1972			0.76)[1]<br>HR=0.70[0.59 0.85][2]<br>HR=0.73[0.60 0.88][3]<br><br>High<br>(males)<br>Death=858<br>HR=0.82[0.74 0.92][1]<br>HR=0.79[0.71 0.89][2]<br>HR=0.77[0.69 0.87][3]<br>(females)<br>Death=284<br>HR=0.78[0.66 0.92][1]<br>HR=0.77[0.66 0.92][2]<br>HR=0.77[0.65 0.91][3]
2	Mok, 2019	Study name: European Prospective Investigation into Cancer (EPIC) study<br><br>Study design: Prospective cohort study<br><br>Follow-up period: 7.6 year (median) 12.0 year follow up for mortality after	n= 14,599<br><br>Inclusion/exclusion: -<br><br>%Females: 56,6%<br><br>Age: baseline 58.8 (SD: 8.8)<br><br>Country: UK<br><br>Type of workers: Working participants of a population recruited through general practitioners practices.	OPA in 4 categories:<br>Unemployed/retired<br>Sedentary work<br>Standing occupation<br>Manual occupation<br><br>Change in OPA in 4x4 categories.<br>-Unemployed<br>-Desk work (sedentary)<br>-Standing<br>-Manual<br><br>Year of exposure	Registers were used to assess mortality from CVD<br><br>ICD 9: 400-438*<br>ICD 10: I10-I79*	[1]Adjusted for age, sex, smoking status, education level, social class, self-rated health, alcohol intake, energy intake, overall diet quality (comprising fruit and vegetables, red and processed meat, fish, wholegrains, refined grains, sweetened confectionery and beverages, ratio of unsaturated to	**Males**<br><br>Sedentary-to-Sedentary:<br>(REF)#<br><br>Sedentary-to-stand<br>HR=0.66 (0.46-0.95)[1] #<br>HR= 0.64 (0.45-0.93)[2] #

		last follow up.		assessment: 1993-1997		saturated fat intake, and sodium) as well as for medical history at baseline (cardiovascular disease, cancer, diabetes, asthma, chronic obstructive pulmonary diseases, and bone fractures), d time-updated variables for smoking, alcohol intake, energy intake, diet quality and medical history at the second clinic visit, as well as period-prevalent heart disease, stroke and cancer from hospital episode statistics up to the final physical activity assessment (third follow-up), body mass index at baseline and at the final physical activity assessment, systolic and diastolic blood pressure, triglycerides, low density lipoprotein cholesterol, and high density lipoprotein cholesterol at baseline and at the second clinic visit<br>[2] Adjusted for above, and for LTPA	Sedentary -to Manual<br>HR=0.66 (0.50-0.88)[1]#<br>HR= 0.67 (0.50-0.89)[2] #<br><br>**Females**<br><br>Sedentary-to-Sedentary<br>(REF)#<br><br>Sedentary-to-stand:<br>HR=0.75 (0.56-1.03)[1]#<br>HR=0.78 (0.57-1.06) #<br><br>Sedentary-to-Manual<br>HR=0.66 (0.40-1.08)[1] #<br>HR= 0.68 (0.41-1.11) #<br><br>(number were based on additional analyses conducted by the authors, which were not reported in the original

							peer-reviewed publication)
3	Chasland; 2017	**<u>Study name:</u>** Busselton Health Survey<br><br>**<u>Study design:</u>** Prospective cohort study<br><br>**<u>Follow-up period:</u>** 20 year	**<u>n</u>**=1649<br><br>**<u>Inclusion/exclusion</u>:** Men aged 20-79 were included, while those who were taking androgens, anti-androgens, or had a history of orchidectomy or prostate cancer, or with missing PA or hormone variables were excluded.<br><br>**<u>%Females</u>: 0**%<br><br>**<u>Age</u>:** 49.8 (15.3)<br><br>**<u>Country</u>:** Australia<br><br>**<u>Type of workers</u>:** Sample of the general population	Participants self-reported how many hours were spend engaging in moderate or vigorous activities at work. Hours per week moderate-intensity activities + 2* hours per week vigorous intensity activities were calculated. Median splits (median 29.0 h/week) were used to assess:<br>• Low occupational PA<br>• High occupational PA<br><br><u>Year of exposure assessment:</u> 1994-1995	CVD mortality was assessed using hospital admission and death records.<br><br>There were 127 (7%) CVD deaths during the follow-up period.<br><br>ICD 9: 390-459*	Age, prevalent CVD, smoking, waist circumference, cholesterol, HDL, lipids medication, diabetes, SBP and hypertension medication.<br><br>Analyses were stratified by testosterone (T), dihidrotestosteron (DHT) and oestradiol (E2) hormones.	**T hormone**<br><u>Low– low hormone</u><br>HR=Reference<br><u>High– low hormone</u><br>HR=0.598 [0.330 1.082]<br><u>Low– high hormone</u><br>HR=1.172 [0.758 1.811]<br><u>High– high hormone</u><br>HR=0.638 [0.336 1.212]<br><br>**DHT hormone**<br><u>Low– low hormone</u><br>HR=Ref<br><u>High– low hormone</u><br>HR=0.640 [0.355 1.151]<br><u>Low– high hormone</u><br>HR=0.836 [0.550 1.271]<br><u>High– high hormone</u><br>HR=0.439 [0.235 0.822]<br><br>**E2 hormone**

							Low– low hormone<br>HR=Ref<br>High– low hormone<br>HR=0.759 [0.389 1.479]<br>Low– high hormone<br>HR=1.620 [1.060 2.476]<br>High– high hormone<br>HR=0.788 [0.422 1.472]<br><br>**All hormones combined**<br>Low<br>HR=Ref #<br>High<br>HR=0.58 [0.37 0.89]#
4	Graff-Iversen; 2007	**Study name:** -<br><br>**Study design:** Prospective cohort study<br><br>**Follow-up period:** 24 years	n=47,405<br><br>**Inclusion/exclusion:** People with CVD or disability pension at baseline or death before 1980 were excluded.<br><br>**%Females**: 23,521 (50%) females and 23,884 males<br><br>**Age:** Mean age in different occupational PA groups ranging from 41.7 to 42.9	Occupational PA was assessed using a questionnaire:<br>• Sedentary (n=5,819 males; n=5,890 females)<br>• Light, demanding much walking (n=5,928 males; n=6,247 females)<br>• Moderately heavy, demanding much walking and	Registers were used to assess mortality from CVD (n=2,563)<br><br>ICD 390-459*	Males and females analyzed separately<br><br>Three models:<br>1. Unadjusted<br>2. Age, county, smoking, and serum total cholesterol.<br>3. Age, county, smoking, serum total cholesterol, education and income.	Sedentary<br>(males)<br>Death=442<br>RR=Ref[1]<br>RR=Ref[2]<br>RR=Ref[3]<br>(females)<br>Death=53<br>RR=Ref[1]<br>RR=Ref[2]<br>RR=Ref[3]<br><br>Light<br>(males)

			**Country:** Norway<br><br>**Type of workers:** General population	occupational lifting (n=2,403 males; n=15,956 females)<br>• Heavy (heavy manual labor; n=3,968 males; n=1,194 females)<br><br>Year of exposure assessment: 1960s			Death=508<br>RR=1.14[1.00 1.30][1]<br>RR=1.10[0.97 1.25][2]<br>RR=1.04[0.91 1.19][3]<br>(females)<br>Death=412<br>RR=1.17[0.88 1.56][1]<br>RR=1.26[0.95 1.69][2]<br>RR=1.19[0.89 1.60][3]<br><br>Moderate<br>(males)<br>Death=503<br>RR=1.13[0.99 1.29][1]<br>RR=1.00[0.87 1.13][2]<br>RR=0.89[0.77 1.03][3]<br>(females)<br>Death=104<br>RR=1.19[0.85 1.66][1]<br>RR=1.21[0.86 1.69][2]<br>RR=1.21[0.86 1.73][3]<br><br>Heavy<br>(males)<br>Death=518

							RR=1.10[0.96 1.24][1]<br>RR=0.97[0.85 1.10][2]<br>RR=0.84[0.73 0.97][3]<br>(females)<br>Death=23<br>RR=0.86[0.53 1.40][1]<br>RR=0.90[0.53 1.53][2]<br>RR=0.87[0.50 1.52][3]
5	Harari; 2015	Study name: CORDIS study<br><br>Study design: Prospective cohort study<br><br>Follow-up period: 22 years	n=4,819<br><br>Inclusion/exclusion: Jewish males were included. Arab males, and females were excluded.<br><br>%Females: 0%<br><br>Age: 42.1 (12.1) years<br><br>Country: Israel<br><br>Type of workers: Industrial workers	Occupational PA was assessed through the question: "Does your work generally entail physical work":<br>• Not at all<br>• Light<br>• Moderate<br>• Hard<br>The first two and last two categories were combined:<br>• Not – light (n=1,870)<br>• Moderate – hard (n=2,949)<br><br>Year of exposure assessment: 1985	Registers were used to assess mortality from IHD (n=170)<br><br>ICD 9: 410-414	Three models:<br>1. Univariate<br>2. Age, socioeconomic status, educational status, father's country of origin, BMI, cholesterol, high-density lipoprotein cholesterol hypertension, diabetes, smoking, coffee, alcohol, maintaining a special diet, and shift work.<br>3. All the above and LTPA	Not - light<br>Death=*51*<br>HR=Ref[1]<br>HR=Ref[2]<br>HR=Ref[3]<br><br>Moderate - hard<br>Death=*119*<br>HR=1.49[1.07 2.07][1]<br>HR=1.35[0.94 1.95][2]<br>HR=1.36[0.94 1.97][3]
6	Holtermann; 2010A (3)	Study name: Copenhagen male study	n=4,943<br><br>Inclusion/exclusion: Participants	"Which description most precisely covers your pattern of	Registers were used to assess	Two models:<br>1. Age	Low<br>Death=123<br>HR=Ref[1]

		Study design:<br>Prospective cohort study<br><br>Follow-up period:<br>30 year	with orthopaedic problems, who were unable to perform the bicycle test, and participants with a history of MI, angina pectoris or intermittent claudication were excluded.<br><br>%Females: 0%<br><br>Age: In different categories ranging from 46.9 (4.8) to 50.8 (5.2) years.<br><br>Country: Denmark<br><br>Type of workers: Employees from 14 companies, covering the railway, public road construction, military, postal, telephone, customs, national bank, and medical industry sectors	physical activity at work?"<br>• Low: mainly sedentary without much walking (n=1,239)<br>• Moderate: Walking quite a bit without carry heavy items (n=2,661)<br>• High: Walking most of the time, walking stairs, lifting items or carry heavy burdens (n=860)<br><br>Strenuous work:<br>"Do you perform strenuous work (work resulting in sweating)?"<br>• Low: seldom or never<br>• Moderate: occasionally<br>• High: often<br><br>Sum score of the two construct<br>• Low physical work demands<br>• Moderate physical work demands<br>• High physical work demands	mortality from IHD (n=86)<br><br>ICD 9: 410-414*	2. Age, BMI, alcohol use, smoking, LTPA, physical fitness	HR=Ref[2]<br><br>Moderate<br>Death=317<br>HR=1.30[1.05 1.60][1]<br>HR=1.26[1.02 1.56][2]<br><br>High<br>Death=588<br>HR=1.69[1.31 2.17][1]<br>HR=1.55[1.19 2.02][2]

				<u>Year of exposure assessment</u>: 1970			
7	Holtermann; 2016	<u>Study name:</u> Copenhagen City Heart Study<br><br><u>Study design:</u> Prospective cohort study<br><br><u>Follow-up period:</u> 18.5 [0.2-20.2] years	<u>n</u>=2534+2190=4724<br><br><u>Inclusion/exclusion</u>: Participants with previous myocardial infarction or stroke were excluded. Participants were exclude if older than the retirement age.<br><br><u>%Females</u>: 2534 (54%)<br><br><u>Age</u>: Average age across categories ranging from 43.0(10.9) to 49.3(11.5) years<br><br><u>Country</u>: Denmark<br><br><u>Type of workers</u>: Sample of the general population	Occupational PA was self-reported with the question: "Which description most precisely covers your pattern of physical activity at work?", with four possible answers:<br>• Mainly sedentary and not much walking at workplace, *e.g.,* desk work, work including assembling of minor parts<br>• Walking around quite a bit at workplace but not have to carry heavy items, e.g. light industrial work, non-sedentary office work, inspection<br>• Most of the time walking, often walking upstairs and lifting various items.<br>• Heavy physical work. Carrying heavy burdens and	National registers were used to ascertain CVD mortality, with 257(10%) deaths<br><br>IDC 10: I00-I99*	Two different models:<br>1. Age and sex<br>2. Age, sex, smoking, BMI, diabetes, household income, alcohol, leisure-time physical activity and cardiorespiratory fitness	<u>Low</u><br>Events=66(5.4%)<br>HR=Ref[1]<br>HR=Ref[2]<br>(females)<br>HR=Ref[1]<br>HR=Ref[2]<br>(males)<br>HR=Ref[1]<br>HR=Ref[2]<br><br><u>Moderate</u><br>Events=83(7.2%)<br>HR=1.15 [0.83 1.59][1]<br>HR=1.18 [0.84 1.65][2]<br>(females)<br>HR=0.97 [0.60 1.58][1]#<br>HR=1.14 [0.69 1.89][2]#<br>(males)<br>HR=1.20 [0.77-1.86][1]#<br>HR=1.21 [0.76 1.90][2]#<br><br><u>High</u><br>Events=108(7.9%)<br>HR=1.75 [1.29 2.39][1]<br>HR=1.56 [1.12 2.16][2]

				carrying out physically strenuous work<br>The highest two categories were combined, yielding three categories:<br>• Low (n=1623)<br>• Moderate (n=1539)<br>• High (n=1562)<br><br><u>Year of exposure assessment:</u> 1991-1994			(females)<br>HR=1.23 [0.74 2.05][1]#<br>HR=1.28 [0.75 2.20][2]#<br>(males)<br>HR=1.88 (1.27-2.77][1]#<br>HR=1.58 [1.04 2.39][2]#
8	Huerta; 2016	<u>Study name:</u> Spanish branch of the European Prospective Investigation into Cancer and Nutrition (EPIC) study<br><br><u>Study design:</u> Prospective cohort study<br><br><u>Follow-up period:</u> 13.6 (1.4) years	<u>n</u>=38.379<br><br><u>Inclusion/exclusion</u>: Participants with baseline ischaemic heart disease, stroke, cancer or asthma at baseline were excluded.<br><br><u>%Females</u>: 23.946 (62%)<br><br><u>Age</u>: Average age across categories ranging from 46.7(8.5) to 50.7(7.3) years<br><br><u>Country</u>: Spain<br><br><u>Type of workers</u>: Sample of the general population	Occupational PA was self-reported using the EPIC physical activity questionnaire in which participants ranked their physical demands as sedentary, standing, manual work, heavy manual work or none. Occupational PA was measured at baseline and on average 3.3 after that to update information:<br>• Sedentary occupation (n=40.798 males; n=23.063 females)<br>• Non-sedentary occupation – standing and manual labour and	CVD mortality was ascertained using records of the Spanish National Statistics Institute.<br><br>ICD 10: I00-I99*	Stratified by gender.<br><br>Adjusted for centre, baseline educational level, height, weight, waist and hip circumferences, self-reported hypertension or hyperlipidemia, diabetes, age, smoking, alcohol, total energy intake, and Mediterranean diet score and other domains of physical activity	**Sedentary occupation**<br>(Males)<br>Cases=29<br>HR=Ref<br>(Females)<br>Cases=3<br>HR=Ref<br><br>**Non-sedentary occupation**<br>(Males)<br>Cases=50<br>HR=0.89[0.55 1.46]<br>(Females)<br>Cases=10<br>HR=1.36 [0.36 5.18]<br><br>**Other (changed job, retired)**

				heavy work (n=78.648 males; n=37.164 females)<br>• Other – changed job, retired (n=52.673 males; n=96.918 females)<br><br><u>Year of exposure assessment:</u> 1992-1996			(Males)<br>Cases=75<br>HR=0.99[0.61 1.61]<br>(Females)<br>Cases=26<br>HR=0.81 [0.23 2.92]
9	Krause; 2017	<u>Study name:</u> Kuopio Ischemic Heart Disease (KIDH) Risk Factor Study<br><br><u>Study design:</u> Prospective cohort study<br><br><u>Follow-up period:</u> 21.7 [0.4 27.8] years	<u>n</u>=1,891<br><br><u>Inclusion/exclusion</u>: Participants who were not working at baseline or in the 12 months prior to baseline were excluded<br><br><u>%Females</u>: 0%<br><br><u>Age</u>: 51.5(5.1) in people without baseline IHD and 53.3(3.9) in people with baseline IHD<br><br><u>Country</u>: Finland<br><br><u>Type of workers</u>: Sample of the general population (ethnically homogeneous)	Interviewers asked subjects in increments of 15 minutes how long they had performed the activities of sitting, standing, walking (on even and uneven surface) and other activities. Using these data, absolute energy expenditure was assessed. Relative energy expenditure was assessed using cardiorespiratory fitness, and was expressed in relative aerobic strain and percent oxygen uptake reserve.<br><br>In models, occupational PA variables were treated as continuous variables while only relative	IHD mortality was ascertained using a national death register.<br><br>661 participants (35% and 16% annual mortality rate) died during the follow-up period, 28% of which due to IHD<br><br>ICD 9: 410-414*	Three models:<br>1. Unadjusted<br>2. Age<br>3. Age, presence in placebo or control group of lipid lowering drug trial, blood glucose, plasma fibrinogen, BMI, blood cholesterol, systolic blood pressure, lipid lowering medication, anti-hypertensive medication, alcohol consumption, smoking, LTPA, income, social support, mental strain and work stress<br>4. All factors from model 3 plus baseline IHD	**Absolute energy expenditure**<br>HR=1.12 [1.05 1.21][1]<br>HR=1.12 [1.04 1.21][2]<br>HR=1.07 [0.98 1.16][3]<br>HR=1.05 [0.96 1.13][4]<br>(No baseline IHD)<br>HR=1.14 [1.03 1.26][4]<br>(Baseline IHD)<br>HR=0.91 [0.79 1.06][4]<br><br>**Relative aerobic strain**<br>HR=1.37 [1.27 1.48][1]<br>HR=1.34 [1.24 1.45][2]<br>HR=1.28 [1.16 1.40][3]

				aerobic strain was also modelled as dichotomous variable:<br>• Low: <33%<br>• High: >33%<br><br>Year of exposure assessment: 1984-1989		Models were also stratified for baseline IHD.	HR=1.23 [1.11 1.35][4]<br>(No baseline IHD)<br>HR=1.30 [1.14 1.49][4]<br>(Baseline IHD)<br>HR=1.19 [1.02 1.38][4]<br><br>Low<br>HR=Ref<br>(without IHD)<br>HR=Ref<br>(with IHD)<br>High<br>HR=1.64 [1.10 2.42][3]<br>(without IHD)<br>HR=1.25 [0.71-2.19][3]<br>(with IHD)<br><br>**Percent oxygen uptake reserve**<br>HR=1.27 [1.19 1.36][1]<br>HR=1.25 [1.17 1.34][2]<br>HR=1.21 [1.12 1.31][3]<br>HR=1.17 [1.08 1.27][4]<br>(No baseline IHD)<br>HR=1.24 [1.10 1.39][4]<br>(Baseline IHD)

							HR=1.14 [1.00 1.30][4]
10	Menotti; 2016	Study name: The Italian Rural areas of the Seven Countries Study of Cardiovascular Diseases<br><br>Study design: Prospective cohort study<br><br>Follow-up period: 50 year	n=1712<br><br>Inclusion/exclusion: -<br><br>%Females: 0%<br><br>Age: 40-59 years<br><br>Country: Italy<br><br>Type of workers: Sample of the general population	Using a questionnaire that matched simple questions with the profession, participants self-reported occupational PA in three categories:<br>• Sedentary<br>• Moderate<br>• Vigorous<br><br>Year of exposure assessment: 1960	CVD mortality was ascertained using death certificates and verbal autopsy. 1669 (97.5%) participants died during the follow up, 43.7% (729) of them due to major CVD causes.<br><br>**ICD code**: not reported in article<br>*CVD mortality was ascertained using;*<br>*CHD, heart disease, stroke, peripheral artery disease, other cardiovascular.*	Age, smoking, Mediterranean diet	Sedentary physical activity<br>HR=Ref<br><br>Moderate physical activity<br>HR=0.87 [0.60 1.28]<br><br>Vigorous physical activity<br>HR=1.01 [0.72 1.41]
11	Moe; 2013 (4)	Study name: Nord-Trøndelag Health Study 2 (HUNT2)<br><br>Study design: Prospective cohort study<br><br>Follow-up period: 12.4 years	n=37,300<br><br>Inclusion/exclusion: Participants with CVD or diabetes were excluded. Stratified analysis on participants with and without metabolic syndrome.<br><br>%Females: 16,217 (51%)	'How would you describe your work?'<br>• Mostly sedentary (eg, at a desk, on an assembly line)<br>• Much walking/lifting at work much walking (eg, delivery work, light	Registers were used to assess mortality from CVD (n=180)<br><br>ICD 9: 390-349*	Age, sex, LTPA, smoking, alcohol, education.	Mostly sedentary<br>*Person years=107,599 (no metabolic syndrome)*<br>Death=55<br>HR=1.19[0.84 1.70]<br>*Person years*=21,134

		(median)	<u>Age</u>: 42.6 (11.5) years in males, 41.9 (10.9) years in females<br><br><u>Country</u>: Norway<br><br><u>Type of workers</u>: Working sample of a general population	industrial work, teaching, postman, nurse, construction work)<br>• Heavy physical work (eg, forestry work, heavy agricultural work, heavy construction work)<br><br><u>Year of exposure assessment:</u> 1995			*(metabolic syndrome)*<br>Death=35<br>HR=2.74[1.82 4.12]<br><br><u>Much walking/lifting</u><br>Person years=230,205 *(no metabolic syndrome)*<br>Death=80<br>HR=Ref<br>*Person years*=37,847 *(metabolic syndrome)*<br>Death=35<br>HR=1.79[1.20 2.66]<br><br><u>Heavy physical work</u><br>Person years=52,698 *(no metabolic syndrome)*<br>Death=45<br>HR=1.20[0.82 1.77]<br>*Person years*=10,549 *(metabolic syndrome)*<br>Death=28	

							HR=3.02[1.93 4.75]
12	Rosengren; 1997	Study name: Multifactor Primary Prevention Study<br><br>Study design: Prospective cohort study<br><br>Follow-up period: 20 years.	n=7,495<br><br>Inclusion/exclusion:-<br><br>%Females:0%<br><br>Age: 47 to 55 years<br><br>Country: Sweden<br><br>Type of workers: general population	Using a questionnaire, occupational PA was graded<br>• Mainly sedentary (n=2,052)<br>• Predominantly walking on one level but no heavy lifting (n=2,814)<br>• Mainly walking, including climbing stairs, or walking uphill or lifting heavy objects (n=1,556)<br>• Heavy physical labor (n=651)<br><br>Year of exposure assessment: 1970	Registers were used to assess mortality from IHD (n=670)<br><br>ICD code: not mentioned	Two models:<br>1. Age<br>2. Age, diastolic blood pressure, serum cholesterols, smoking, alcohol abuse, BMI, diabetes, working class	Sedentary work<br>Death=183<br>RR=Ref[1]<br>RR=Ref[2]<br><br>Light mobile<br>Death=280<br><br>Heavy work<br>Death=145<br><br>Very heavy, strenuous work<br>*All-cause mortality*<br>Death=64<br>RR=1.05[0.83 1.32][1]<br>RR=0.83[0.62 1.12][2]
13	Smigielski; 2016	Study name: National Multicentre Health Survey (WOBASZ)<br><br>Study design: Prospective cohort study<br><br>Follow-up period: 39.4 months [2.9 69.2]	n=3,577 (occupational PA was only analysed for the youngest age group of 50-59 years; n=1,690)<br><br>Inclusion/exclusion: Only participant aged 50 or older were included<br><br>%Females: 0%<br><br>Age: 61.9 (8.5%)<br><br>Country: Poland	Self-reported occupational PA with high occupational PA defined as intensive physical work during at least half of the overall work time:<br>• Low (n=1198)<br>• High (n=492)<br><br>Year of exposure assessment: 2003-2005	CVD mortality was assessed through electronic registers.<br><br>150 (out of the total sample) participants (4%) died during the follow-up period, with 14, 15 and 14 from IHD,	Both models are stratified by age groups.<br><br>Two models:<br>1. Univariate<br>2. Multivariate: leisure-time and commuting physical activity, place of living, age, smoking, blood pressure, BMI, HDL, homocysteine and diabetes.	Low<br><br>High<br>Deaths=4<br>mortality rate=0.81<br><br>Beta (SE)=–1.201(0.530)[1]<br>HR=0.30[1]<br>p-val=0.024[1]<br><br>Beta (SE)=–1.007 (0.538)[2]

			Type of workers: Sample of the general population		cerebrovascular and capillary disease, respectively<br><br>ICD: not mentioned.		HR=0.365[2]<br>p-val=0.061[2]
14	Stamatakis; 2013	Study name: Health Survey for England (HSE) and the Scottish Health Survey (SHS)<br><br>Study design: Prospective cohort study<br><br>Follow-up period: 12.9 year (mean)	n=11,168<br><br>Inclusion/exclusion: -<br><br>%Females: 5,380 (48%)<br><br>Age: Mean age in various groups ranging from 49.2 (6.6) to 51.2 (7.7)<br><br>Country: UK<br><br>Type of workers: Working sample of general population	Using the question ‘‘When you’re at work are you mainly”, Occupational PA was assessed, categorized into:<br>• Sitting down (n=2,090 females; n=2,328 males)<br>• Standing up or walking (n=3,124 females; n=3,237 males)<br><br>Year of exposure assessment: 1994	Register data were used to assess mortality from CVD (n=177)<br><br>ICD 9: 390-459<br>ICD 10: I01-I99	Three models:<br>1. Age<br>2. Age, waist circumference, general health, psychological health, alcohol, smoking, non-occupational PA, prevalent CVD at baseline, prevalent cancer at baseline<br>3. All the above, and occupational social class and age finished educations.	Sitting<br>(females)<br>Death=11<br>HR=Ref[1]<br>HR=Ref[2]<br>HR=Ref[3]<br>(males)<br>Death=51<br>HR=Ref[1]<br>HR=Ref[2]<br>HR=Ref[3]<br><br>Standing/walking about<br>(females)<br>Death=31<br>HR=0.63[0.82 3.25][1]<br>HR=1.74[0.86 3.51][2]<br>HR=1.53[0.72 3.24][3]<br>(males)<br>Death=84<br>HR=1.03[0.73 1.46][1]<br>HR=0.99[0.69 1.41][2]<br>HR=0.98[0.66 1.45][3]

15	Yu; 2003 (5)	Study name:<br>Caerphilly collaborative heart disease study<br><br>Study design:<br>Prospective cohort study<br><br>Follow-up period:<br>10.5 year	n=1,975<br><br>Inclusion/exclusion: Participants with existing IHD, a diagnosed MI, angina, participants who died within 2 years from baseline were excluded<br><br>%Females: 0%<br><br>Age: 45–59 years<br><br>Country: UK<br><br>Type of workers: General population	Using a self-administered questionnaire, occupational PA was assessed and categorised into quartiles.<br><br>Year of exposure assessment: 1984	Registers were used to assess mortality from:<br>• CVD (n=111)<br>IHD (n=82)<br><br>ICD 9: 390-358*	Two models<br>1. Age<br>2. Age, diastolic blood pressure, and BMI, smoking, social class, family history of IHD among first degree relatives before age 55, history of diabetes mellitus in the past five years, LTPA and employment status	Quartile 1<br>*CVD mortality*<br>HR=Ref[1]<br>HR=Ref[2]<br>*IHD mortality*<br>HR=Ref[1]<br>HR=Ref[2]<br><br>Quartile 2<br>*CVD mortality*<br>HR=1.42[0.85 2.37][1]<br>HR=1.59[0.93 2.71][2]<br>*IHD mortality*<br>HR=1.46[0.82 2.62][1]<br>HR=1.68[0.92 3.08][2]<br><br>Quartile 3<br>*CVD mortality*<br>HR=1.68[1.00 2.80][1]<br>HR=1.75[1.00 3.06][2]<br>*IHD mortality*<br>HR=1.32[0.71 2.45][1]<br>HR=1.43[0.73 2.79][2]<br><br>Quartile 4<br>*CVD mortality*<br>HR=0.87[0.48 1.59][1]<br>HR=0.73[0.38

							1.41][2]<br>*IHD mortality*<br>HR=0.94[0.48 1.84][1]<br>HR=0.80[0.38 1.69][2]
16	Hayashi, 2016	Study name<br>JACC Study (1988-1990. Until 2009<br>Study design:<br>Cohort<br>Follow up period :<br>19,2 years	N= 110.585<br><br>% Females;<br>58%<br><br>Age;<br>40-79 years<br><br>Type of workers:<br>Population based sample of Japanese residents.<br><br>County;<br>Japan	"During your working life, until the present, which OPA have you been involved in?": 1) mostly sitting, 2) sitting and standing, 3) mostly standing, and 4) standing and walking. Therefore, OPA was categorized into four groups: mostly sitting, sitting/standing, mostly standing, and standing/walking.	Mortality data were then centralized at the Ministry of Health, Labour and Welfare, and the underlying causes of death were coded for National Vital Statistics<br><br>ICD 10: I01-I99*	Model 1: adjusted for age and sex<br>Model 2: adjusted as in model 1 and for body mass index, history of hypertension , history of diabetes mellitus, alcohol intake , smoking, education level, high perceived mental stress, hours of exercise, hours of walking, and hours of TV watching.<br>Model 3: adjusted as model 2 and for employment status.	All participants;<br><br>Mostly sitting;<br>REF<br><br>Sitting and standing<br>HR: 1.03 (0.93-1.13) [1]<br>HR: 1.04 (0.94-1.15) [2]<br>HR: 1.04 (0.94-1.15) [3]<br><br>Mostly standing<br>HR: 1.21 (1.05-1.38) [1]<br>HR: 1.21 (1.05-1.39) [2]<br>HR: 1.20 (1.05-1.38) [3]<br><br>Standing and walking<br>0.97 (0.90-1.05) [1]<br>0.99 (0.91-1.07) [2]<br>0.98 (0.90-1.07) [3]<br><br>Men:

								<u>Sitting and standing:</u><br>HR: 0.99 (0.87-1.14) [1]<br>HR: 1.02 (0.89 - 1.17) [2]<br>HR: 1.01 (0.88-1.16) [3]<br><br><u>Mostly standing:</u><br>HR: 1.19 (0.99-1.42) [1]<br>HR: 1.20-1.00-1.45) [2]<br>HR: 1.19 (0.99-1.43) [3]<br><br><u>Standing and walking:</u><br>HR: 1.02 (0.92-1.13) [1]<br>HR: 1.05 (0.94-1.17) [2]<br>HR: 1.03 (0.93-1.15) [3]<br><br><u>Women</u><br><br><u>Sitting and standing</u><br>HR: 1.03 (0.89-1.19) [1]<br>HR: 1.05 (0.91-1.22) [2]<br>HR: 1.05 (0.91-1.22) [3]

							Mostly standing:<br>HR: 1.22 (0.99-1.50) [1]<br>HR: 1.24 (1.01-1.53) [2]<br>HR: 1.25 (1.01-1.54) [3]<br><br>Standing and walking:<br><br>HR: 0.89 (0.79-1.01)[1]<br>HR: 0.91 (0.81-1.4) [2]<br>HR: 0.91 (0.80-1.04) [3]
17	Hermansen, 2019	Study name:<br>The Finnmark study<br><br>Study design:<br>Longitudinal population based study.<br><br>Follow up period:<br>23.3 years	N= 17.697<br><br>Inclusion/exclusion<br><br>%Females.<br>49.4%<br>Age:<br>47.2 (9.2)<br>Country<br>Norway<br>Type of workers:<br>Population-based cohort in the Finnmark county in Northern Norway.	OPA was assessed by the Saltin-Grimby Physical Activity Level Scale21 with four mutually exclusive options: 'mostly sedentary', 'walking' (e.g. shop assistant, light industrial work, education), 'walking and lifting' (e.g. mailman, heavy industrial work, construction work) and 'heavy manual labour' (e.g. forestry work, heavy agriculture work,	Date and cause of death were registered. CVD death was defined using the underlying cause of death.<br><br>ICD-9: 390– 459<br>ICD-10: I00–I99	Model 1:<br>Adjusted for age, sex, smoking status, BMI and LTPA.<br><br>Model 2:<br>Age, sex, smoking status, BMI, self-reported angina pectoris, myocardial infarction, cerebral insult, diabetes, anti-hypertensive medication and leisure time physical activity.	Model:<br><br>Mostly sedentary<br>HR: 1.26 (1.10-1.45)[1]<br>HR: 1.17 (1.02-1.34)[2]<br><br>Walking<br>HR: 1.18 (1.02-1.35)[1]<br>HR: 1.09 (1.02-1.34)[2]<br><br>Walking and lifting:<br>REF

				heavy construction work).<br><br><u>Year of exposure assessment</u>:<br>data from the third Finnmark Study in 1987–1988,			Heavy manual labour<br>HR: 1.14 (0.95-1.36) [1]<br>HR: 1.15 (0.96-1.38) [2]
18	Wanner, 2019	<u>Study name:</u><br>1: NRP1A<br><u>Study design:</u><br>Longitudinal population based.<br><u>Follow up period:</u>	<u>N=</u><br>1. 2818 Men – 1578 Women<br><br><u>Inclusion/exclusion</u><br><br><u>%Females.</u><br>1. 36%<br><u>Age:</u><br>1. Men = 40.7 Women = 38.1<br><br><u>Country</u><br>Switzerland<br><u>Type of workers:</u><br>Population based sample	NRP1A:<br>**Low:** Sedentary, taking stairs of less than five floors or walking less than 800m per day at work<br>**Moderate:**<br>Taking stairs of 5-20 floors or walking 800m to 3 km per day at work<br>**High:** Taking stairs of more than 20 floors or walking more than 3 km per day at work<br><br>**Assessment data:**<br><br>The NRP1A study was conducted from 1977 to 1979	Linking the studies with the Swiss National Cohort established a mortality follow-up until 2015.<br><br>ICD 8: 390-448<br>ICD 10: I00-I99	<u>Models adjusted for:</u><br>Age, education, nationality, marital status, smoking, regular meals, active commuting, LTPA, sport activities, BMI, blood pressure, and blood cholesterol	**<u>NRP1A</u>**<br><br>**<u>MEN</u>**<br><u>Low:</u><br>REF<br><u>Moderate</u><br>HR: 1,00 (0.80-1.26)<br><u>High</u><br>HR: 0.88 (0.63-1.22)<br><br>**WOMEN:**<br><u>Low:</u><br><u>REF</u><br><u>Moderate:</u><br>HR: 0.90 (0.59-1.36)<br><u>High:</u><br>HR: 1.13 (0.54-2.34)
19	Wanner, 2019	<u>Study name:</u><br>2: MONICA	<u>N=</u><br>2. 3988 men – 1792 women	MONICA:<br>**Low:** Mostly sitting	Linking the studies with the Swiss National	<u>Models adjusted for:</u><br>Age, education, nationality, marital	 **<u>MONICA:</u>**<br>**MEN**

		Study design:<br>Longitudinal population based.<br><br>Follow up period:	Inclusion/exclusion<br><br>%Females.<br>2. 31%<br>Age:<br>2. Men = 45.2<br>Women= 43.2<br><br>Country<br>Switzerland<br>Type of workers:<br>Population based sample	**Moderate:** Mostly standing and walking frequently, but not lifting or carrying heavy objects<br>**High:** Often having to take stairs and carrying light objects<br>**High:** A lot of physical effort and frequently carrying heavy objects<br><br>the MONICA study were conducted in Switzerland between 1984 and 1993.	Cohort established a mortality follow-up until 2015.<br><br>ICD 8: 390-448<br>ICD 10: I00-I99	status, smoking, regular meals, active commuting, LTPA, sport activities, BMI, blood pressure, and blood cholesterol	Low:<br>REF<br>Moderate:<br>HR: 1.41 (1.03-1.91)<br>High:<br>HR: 1.08 (0.76-1.52)<br><br>**WOMEN:**<br>Low:<br>REF<br>Moderate:<br>HR: 0.64 (0.29-1.40)<br>High:<br>HR: 1.88 (0.84-4.20)
20	Bennett, 2017	Study name:<br>China Kadoorie Biobank<br><br>Study design:<br>Prospective study<br><br>Follow-up period:<br>7.5 year.	n= 487.334<br>OPA= 347.84 #<br><br>Inclusion/exclusion:<br>Individuals with prior physician-diagnosed IHD (n = 15 472), those who had experienced stroke/transient ischemic attack (n = 8884), and those reporting implausibly small, large, or conflicting levels of physical activity (n = 10201) were excluded<br><br>%Females: 59.1%<br><br>Age:<br>35-74 years	The CKB physical activity questionnaire covered relevant questions on the intensity, frequency, and time spent on occupational tasks, commuting, household tasks, and leisure time activities. Metabolic equivalents of tasks (METs) of different types of activities were adopted from the 2011 compendium of physical activities. MET of each activity was multiplied by the	Vital status of participants was monitored periodically through China's nationally representative (DSP) death registries.<br><br>The causes of death were coded by trained DSP staff<br><br>By January 1, 2015, 25488 participants (5%)	All HRs are stratified by age, sex, and region and adjusted for income, education, BMI, alcohol, smoking, SBP, fresh fruit intake, sedentary leisure time, and self-rated health. Occupational physical activity models were additionally adjusted for non-occupational physical activity.	**Occupational MET-h/day**<br><br>**0.1-5.9**<br>REF<br>**6.0-13.8**<br>HR: 0.84 (0.79-0.89)#<br>**13.9-25.7**<br>HR: 0.78 (0.73-0.83)#<br>**>28.8**<br>HR: 0.76 (0.70-0.82)#

			Mean; 51.1 (10.5)<br><br>Country: China<br><br>Type of workers: Representative sample of inhabitants of 5 rural and urban Chinese areas.	frequency and duration of PA to calculate PA in MET hours per day (MET- h/ day) from each activity Occupational physical activity included all physical activity that was performed during paid employment.<br><br>Year of exposure assessment:<br>between June 2004 and July 2008.	had died and 2411 (0.4%)were lost to follow-up.<br><br>ICD: I00-I99		
21	Mikkola, 2019	Study name:<br>Helsinki Birth cohort study<br><br>Study design:<br>Longitudinal population based study.<br><br>Follow up period:<br>26 years	N= 13.345<br><br>Inclusion/exclusion<br><br>%Females.<br>47,5%<br><br>Age:<br>45-54<br><br>Country<br>Finland.<br><br>Type of workers:<br>Population-based sample of residents in the Helsinki area.	The development of the two physical work characteristics (physical heaviness of work and sitting at work) was based on two distinct questions in the aforementioned survey: (1) ‘Does your current job involve heavy physical work, in which you have to lift or carry heavy items, to dig, shovel or pound?’ (yes/no) and<br><br>Physical heaviness of work and sitting at work were categorised according to gender-specific quartiles. The quartiles of physical	Mortality<br>Dates and causes of death were obtained from the Finnish National Death Register.<br><br>Cause of death was based on the primary cause of death in the register.<br><br>ICD 9: 400-499<br>ICD 10: I00-I99	Model 1:<br>Adjusted for Age and years of education	**Women**<br>**Model 1:**<br><br>Lowest: (quartile 1)<br>REF<br><br>Quartile 2:<br>HR: 1.05 (0.67-1.65) #<br><br>Quartile 3:<br>HR: 1,08 (0.69-1.68) #<br><br>Quartile 4:<br>HR: 1.34 (0.90-2.00) #<br><br>**Men**<br>**Model 1:**

				heaviness of work were ≤1.10, 1.11–5.30, 5.31–21.70 and ≥21.71 among women and ≤3.40, 3.41– 12.50, 12.51–44.0 and ≥44.01 among men.<br><br>Assessment year:<br>1990			Quartile 1:<br>REF<br>Quartile 2:<br>HR: 1,14(0.85-1.53) #<br><br>Quartile 3:<br>HR: 1.39 (1.06-1.81) #<br><br>Quartile 4:<br>HR: 1.70 (1.30-2.23) #
22	Bahls, 2018	Study name:<br>The Study of Health in Pomerania (SHIP)<br><br>Study design:<br>Prospective population- based cohort of adults<br><br>Follow up period:<br>11.5 years (25th and 75th percentile: 10.7-12.4)	N= 2,935<br><br>Inclusion/exclusion<br><br>%Females.<br>45.2<br><br>Age:<br>51 years (39-62)<br><br>Country<br>Germany<br><br>Type of workers:<br>Population based study in Pomerania area.	Domain specific physical activity was assessed using the well-established Baecke questionnaire. questions are scored on a five-point Likert scale, ranging from never to always or very often<br>Work Physical activity<br>WPA (i.e. one's own WPA compared to others of similar age followed by questions regarding sitting, standing, walking, lifting of heavy loads and sweating at work as well as if one is tired after work)<br><br>Assessment year:<br>1997-2001	Death certificates were requested from the local health authorities and were coded by a certified nosologist according to the International Classification of Diseases<br><br>ICD 10: I10-I79	**Model 1:**<br>Unadjusted<br><br>**Model 2:**<br>included age, sex, years of schooling, income, smoking, body mass index in	**linear regression coefficients per standard deviation increase.**<br><br>HR: 1.29 (0.51-3.28) [2]<br><br>**Dichotomous:**<br><br>**WPA: <2.6**<br>REF<br><br>**WPA: >2.5**<br>HR: 1.27 (0.28-5.67)[1] #<br><br>HR: 1.72 (0.35-8.51) [2] #

23	Bahls, 2018	Study name:<br>Cardiovascular Disease, Living and Ageing in Halle Study<br><br>Study design:<br>Prospective population- based cohort of adults<br><br>Follow up period:<br>8.2 years (25th- 75th :percentile 7.4-9.2	N= 1.779<br><br>Inclusion/exclusion<br><br>%Females.<br>59.8%<br><br>Age:<br>64 (56;73)<br><br>Country<br>Germany<br><br>Type of workers:<br>Population based study in Halle area	Domain specific physical activity was assessed using the well-established Baecke questionnaire. questions are scored on a five-point Likert scale, ranging from never to always or very often<br>Work Physical activity<br>WPA (i.e. one's own WPA compared to others of similar age followed by questions regarding sitting, standing, walking, lifting of heavy loads and sweating at work as well as if one is tired after work)<br><br>Assessment year:<br>2002-2006	Death certificates were requested from the local health authorities and were coded by a certified nosologist according to the International Classification of Diseases<br><br>ICD 10: I10-I79	**Model 1:**<br>Unadjusted<br><br>**Model 2:**<br>included age, sex, years of schooling, income, smoking, body mass index	**linear regression coefficients per standard deviation increase.**<br><br>HR: 2.24 (0.57-8.84)<br><br><br>**WPA: <2.6**<br>REF<br><br>**WPA: >2.5**<br>HR: 1.82 (0.33-9.92) [1]#<br><br>HR: 2.32 (0.32-14.63)[2]#

OPA = occupational physical activity, LTPA = Leisure time physical activity, SES = socio-economic status, BMI = body mass index.

***= International Classification of Diseases and Related Health Problem.**

- **ICD 9: World Health Organization. (1978). International classification of diseases : [9th] ninth revision, basic tabulation list with alphabetic index.**
- **ICD 10: World Health Organization. (2004). ICD-10 : international statistical classification of diseases and related health problems : tenth revision, 2nd ed. World Health)**

#=Data obtained after re-analysis of study data by authors.

Table S7: risk of bias assessment:

	First author, Year;	Item 1	Item 2	Item 3	Item 4	Item 5a	Item 5b	Item 6	Item 7	Item 8	Item 9	Item 10	Item 11	Sum score
1	Barengo 2004 [3]	2	2	2	2	1	2	2	2	2	2	2	2	0,96
2	Besson 2008 [4]	2	2	1	2	2	2	1	2	2	2	1	2	0,88
3	Chasland; 2017	2	2	2	2	1	2	2	1	2	2	2	1	0,88
4	Graff-Iversen 2007 [5]	2	2	1	2	2	2	2	1	2	1	2	2	0,88
5	Harari 2015 [6]	2	2	2	2	1	2	2	2	2	2	2	2	0,96
6	Holtermann 2010A [7]	2	2	2	2	2	2	2	2	2	1	2	2	0,96
7	Holtermann; 2017	2	2	2	2	2	2	2	2	2	2	2	2	1,00
8	Huerta; 2016	2	2	2	2	1	2	2	2	2	2	2	2	0,96
9	Krause; 2017	2	2	2	2	2	2	2	2	2	2	2	2	1,00
10	Menotti; 2016	2	2	2	2	1	1	2	2	2	2	2	2	0,92
11	Moe 2013 [10]	2	2	2	2	2	2	1	2	2	2	2	2	0,96
12	Rosengren 1997 [11]	2	2	1	2	0	2	1	1	2	1	1	2	0,71
13	Smigielski; 2016	2	2	2	2	1	1	2	1	2	2	1	2	0,83
14	Stamatakis 2013 [12]	2	2	2	2	1	2	2	2	2	2	2	2	0,96
15	Yu 2003 [13]	2	2	2	1	1	2	1	2	2	2	1	1	0,79
16	Bennet, 2017	2	2	2	2	2	2	2	2	2	2	2	1	0,96
17	Bahls, 2018	2	2	1	2	1	2	0	2	2	1	1	2	0,75
18	Hermansen, 2019	2	2	2	2	2	1	2	2	2	2	2	2	0,96
19	Mikkola, 2019	2	2	2	2	1	2	2	2	2	1	2	2	0,92
20	Wanner, 2019	2	2	1	1	2	2	2	2	2	2	2	2	0,92
21	Hayashi, 2016	2	2	2	2	2	2	2	2	2	2	2	2	1,00

mean	0,91
SD	0,08
min	0,71
max	1,00

Table S8: References table 1.

Increments of OPA	Reference
Highest vs lowest OPA category	(6-26)
- Males	(7,9-12, 14-19, 21-26)
- Females	(7,10,12,15-16,19,21,24-26)
**Type of mortality**	
- Overall cardiovascular mortality	(6-10,12-13,15-16,18-21,23-26)
- Ischaemic heart disease mortality	(11,14,17,25,26)
**OPA measurement**	
Quantitative self-rated measurements (e.g. amount of stairs/hours/frequency)	(7,8,16,25,26)
Qualitative self-rated measurements (e.g. non quantifiable exposure or exertion)	(5,9-15,17-25)
**Follow up duration**	
- Between 3.3- 13.6 years	(6,8,16,20,23,24,26)
- Between 15.9- 21.7 years	(7,9,12,15,17,21,23)
- Between 22 – 50 years	(10,11,13,14,18,19,25)
**Year of baseline assessment**	
- Before 1989	(7,10,11,12,13,14,17,18,23,25,26)
- 1990 and later	(6,8,9,15,16,19,20,21,23,24)
**Sample size of study**	
- 10.000 or less	(5,9,11,14,16,17,18,21,22,23,25,26))
- >10.000	(7,8,10,12,13,16,20,24)
**Adjusted for total or leisure-time physical activity.**	
- Yes	(5-8, 11-17, 20, 21, 23-26)
- No	(9,10,18,19,23)
**Adjusted for socioeconomic status (education or social class)**	
- Yes	(6-8, 10-12, 19-21, 24-26)
- No	(9, 13-14, 18, 22, 23)
**Adjusted for BMI (or waist circumference)**	
- Yes	(6-9, 11-17, 21-26)
- No	(10, 18-20)
**Adjusted for gender, age, LTPA, SES and BMI**	
- Yes	(6-8, 11, 12, 15-17, 21, 24-26)
- No	(9-10, 13-14, 18-20, 22-23)
**Adjustment for diet (Energy intake/ Mediterranean diet)**	
- Yes	(11-16-18-21)
- No	(6-10, 12-16, 17, 19-20, 23-26)

Figure S1: Risk of bias;

Publication bias for the main analyses of 23 studies assessing 33 estimates.

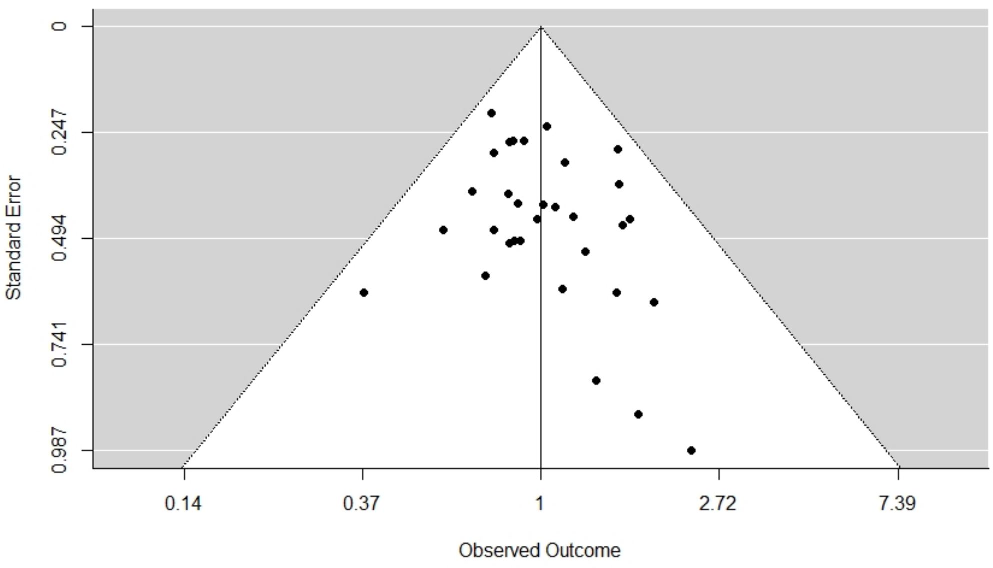


```
test for funnel plot asymmetry: z = 1.1903, p = 0.2339
Rank Correlation Test for Funnel Plot Asymmetry
Kendall's tau = 0.1804, p = 0.1409
```

Figure S2: Forest plot

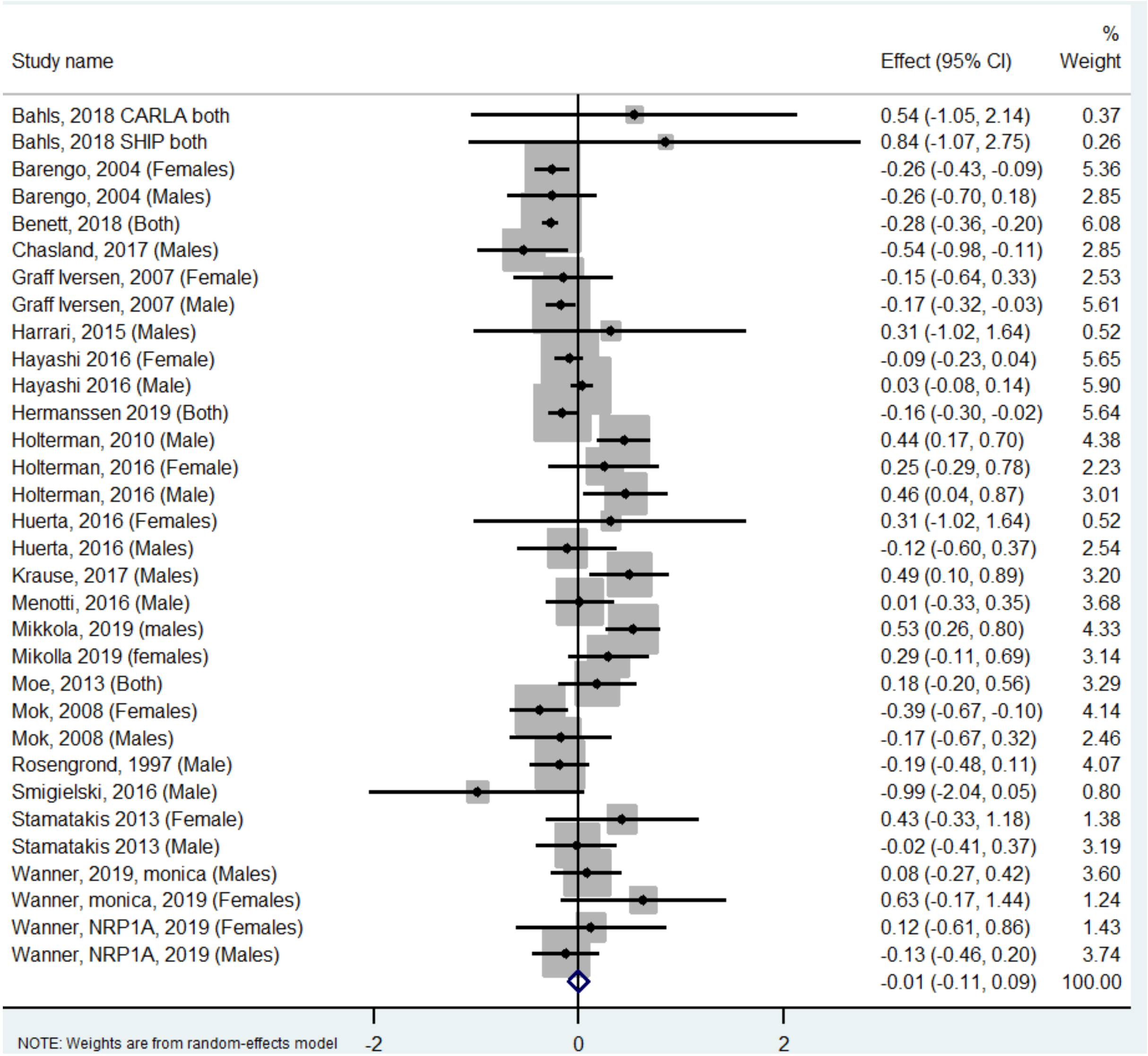

Supplement text A : Does the literature support the physical activity health paradox?

Our current findings on occupational physical activity and CVD mortality with HRs near one and wide confidence intervals straddling zero effects in both directions are consistent and compatible with the hypothesis of different health effects of occupational physical activity and leisure time physical activity, as postulated by the 'physical health activity paradox'(27, 28). Furthermore, our diagnosis-specific findings of a 15% increased risk in IHD mortality, albeit not statistically significant but with confidence intervals (0.88-1.47), favouring detrimental over any null or possible protective effects. Providing additional empirical support for the hypothesis that high levels of occupational physical activity may be responsible for higher IHD mortality risks among manual workers. These findings also support the observed relatively lower reductions in IHD mortality during past decades among occupational groups that continued to be exposed to heavy physical job demands(29, 30) and thus ultimately for the positive association between occupational physical activity and all-cause mortality(31). The fact that most studies adjusted for key behavioural cardiovascular risk factors and the subgroup analysis by SES do not support the notion that detrimental effects of high occupational physical activity are fully attributable to those behavioural factors or any other SES-related factors other than occupational physical activity itself. These findings of our review therefore provide additional empirical support for the physical activity health paradox.

1. Sedentary Behaviour Research Network. Letter to the editor: Standardized use of the terms "sedentary" and "sedentary behaviours". Applied Physiology, Nutrition, and Metabolism. 2012;37(3):540-2.
2. Tremblay MS, Colley RC, Saunders TJ, Healy GN, Owen N. Physiological and health implications of a sedentary lifestyle. Applied Physiology, Nutrition, and Metabolism. 2010;35(6):725-40. Epub 2010/12/18. doi: 10.1139/H10-079. PubMed PMID: 21164543.
3. Holtermann A, Mortensen OS, Burr H, Søgaard K, Gyntelberg F, Suadicani P. Physical demands at work, physical fitness, and 30-year ischaemic heart disease and all-cause mortality in the Copenhagen Male Study. Scandinavian Journal of Work, Environment & Health. 2010;36(5):357-65. PubMed PMID: 20352174.
4. Moe B, Mork PJ, Holtermann A, Nilsen TI. Occupational physical activity, metabolic syndrome and risk of death from all causes and cardiovascular disease in the HUNT 2 cohort study. Occupational and Environmental Medicine. 2013;70(2):86-90. doi: 10.1136/oemed-2012-100734. PubMed PMID: 23022656.
5. Yu S, Yarnell JW, Sweetnam PM, Murray L, Caerphilly s. What level of physical activity protects against premature cardiovascular death? The Caerphilly study. Heart. 2003;89(5):502-6. PubMed PMID: 12695452; PubMed Central PMCID: PMC1767647.
6. Bahls M, Gross S, Baumeister SE, Volzke H, Glaser S, Ewert R, et al. Association of domain-specific physical activity and cardiorespiratory fitness with all-cause and cause-specific mortality in two population-based cohort studies. Scientific reports. 2018;8(1):16066. Epub 2018/10/31. doi: 10.1038/s41598-018-34468-7. PubMed PMID: 30375472; PubMed Central PMCID: PMCPMC6207740.
7. Barengo NC, Hu G, Lakka TA, Pekkarinen H, Nissinen A, Tuomilehto J. Low physical activity as a predictor for total and cardiovascular disease mortality in middle-aged men and women in Finland. European heart journal. 2004;25(24):2204-11. Epub 2004/12/14. doi: 10.1016/j.ehj.2004.10.009. PubMed PMID: 15589637.
8. Bennett DA, Du H, Clarke R, Guo Y, Yang L, Bian Z, et al. Association of Physical Activity With Risk of Major Cardiovascular Diseases in Chinese Men and Women. JAMA cardiology. 2017;2(12):1349-58. Epub 2017/11/09. doi: 10.1001/jamacardio.2017.4069. PubMed PMID: 29117341; PubMed Central PMCID: PMCPMC5814992.
9. Chasland L, Knuiman M, Divitini M, Chan Y, Handelsman D, Naylor L, et al. Physical activity level and androgen concentrations are independently and additively associated with lower cardiometabolic risk in men. Heart Lung and Circulation. 2017;26:S347-S8. doi: 10.1016/j.hlc.2017.06.707.
10. Graff-Iversen S, Selmer R, Sørensen M, Skurtveit S. Occupational physical activity, overweight, and mortality: a follow-up study of 47,405 Norwegian women and men. Research quarterly for exercise and sport. 2007;78(3):151-61. Epub 2007/08/08. doi: 10.1080/02701367.2007.10599412. PubMed PMID: 17679488.
11. Harari G, Green MS, Zelber-Sagi S. Combined association of occupational and leisure-time physical activity with all-cause and coronary heart disease mortality among a cohort of men followed-up for 22 years. Occupational and environmental medicine. 2015;72(9):617-24. Epub 2015/03/26. doi: 10.1136/oemed-2014-102613. PubMed PMID: 25805756.
12. Hayashi R, Iso H, Cui R, Tamakoshi A. Occupational physical activity in relation to risk of cardiovascular mortality: The Japan Collaborative Cohort Study for Evaluation for Cancer Risk (JACC Study). Preventive medicine. 2016;89:286-91. Epub 2016/06/18. doi: 10.1016/j.ypmed.2016.06.008. PubMed PMID: 27311336.
13. Hermansen R, Jacobsen BK, Lochen ML, Morseth B. Leisure time and occupational physical activity, resting heart rate, and mortality in the Arctic region of Norway. The Finnmark study. European journal of preventive cardiology. 2019;26:S169. PubMed PMID: rayyan-63656434.
14. Holtermann A, Mortensen OS, Burr H, Søgaard K, Gyntelberg F, Suadicani P. Physical demands at work, physical fitness, and 30-year ischaemic heart disease and all-cause mortality in the

Copenhagen Male Study. Scandinavian journal of work, environment & health. 2010;36(5):357-65. Epub 2010/03/31. doi: 10.5271/sjweh.2913. PubMed PMID: 20352174.
15. Holtermann A, Marott JL, Gyntelberg F, Sogaard K, Mortensen OS, Prescott E, et al. Self-reported occupational physical activity and cardiorespiratory fitness: Importance for cardiovascular disease and all-cause mortality. Scandinavian journal of work, environment & health. 2016;42(4):291-8. Epub 2016/04/22. doi: 10.5271/sjweh.3563. PubMed PMID: 27100403.
16. Huerta JM, Chirlaque MD, Tormo MJ, Buckland G, Ardanaz E, Arriola L, et al. Work, household, and leisure-time physical activity and risk of mortality in the EPIC-Spain cohort. Preventive medicine. 2016;85:106-12. Epub 2016/02/11. doi: 10.1016/j.ypmed.2016.02.009. PubMed PMID: 26861751.
17. Krause N, Arah OA, Kauhanen J. Physical activity and 22-year all-cause and coronary heart disease mortality. American journal of industrial medicine. 2017;60(11):976-90. Epub 2017/09/25. doi: 10.1002/ajim.22756. PubMed PMID: 28940659.
18. Menotti A, Puddu PE, Maiani G, Catasta G. Cardiovascular and other causes of death as a function of lifestyle habits in a quasi extinct middle-aged male population. A 50-year follow-up study. International journal of cardiology. 2016;210:173-8. Epub 2016/03/11. doi: 10.1016/j.ijcard.2016.02.115. PubMed PMID: 26962972.
19. Mikkola TM, von Bonsdorff MB, Salonen MK, Kautiainen H, Ala-Mursula L, Solovieva S, et al. Physical heaviness of work and sitting at work as predictors of mortality: a 26-year follow-up of the Helsinki Birth Cohort Study. BMJ open. 2019;9(5):e026280-e. doi: 10.1136/bmjopen-2018-026280. PubMed PMID: 31101697.
20. Moe B, Mork PJ, Holtermann A, Nilsen TI. Occupational physical activity, metabolic syndrome and risk of death from all causes and cardiovascular disease in the HUNT 2 cohort study. Occupational and environmental medicine. 2013;70(2):86-90. Epub 2012/10/02. doi: 10.1136/oemed-2012-100734. PubMed PMID: 23022656.
21. Mok A, Khaw K-T, Luben R, Wareham N, Brage S. Physical activity trajectories and mortality: population based cohort study. BMJ. 2019;365:l2323. doi: 10.1136/bmj.l2323.
22. Rosengren A, Wilhelmsen L. Physical activity protects against coronary death and deaths from all causes in middle-aged men: Evidence from a 20-year follow-up of the primary prevention study in Göteborg. Annals of Epidemiology. 1997;7(1):69-75. doi: https://doi.org/10.1016/S1047-2797(96)00106-8.
23. Śmigielski J, Ruszkowska J, Piotrowski W, Polakowska M, Bielecki W, Hanke W, et al. The relationship between physical activity level and selected cardiovascular risk factors and mortality of males ≤ 50 years in Poland - the results of follow-up of participants of National Multicenter Health Survey WOBASZ. International journal of occupational medicine and environmental health. 2016;29(4):633-48. Epub 2016/07/23. doi: 10.13075/ijomeh.1896.00660. PubMed PMID: 27443759.
24. Stamatakis E, Chau JY, Pedisic Z, Bauman A, Macniven R, Coombs N, et al. Are sitting occupations associated with increased all-cause, cancer, and cardiovascular disease mortality risk? A pooled analysis of seven British population cohorts. PLoS One. 2013;8(9):e73753-e. doi: 10.1371/journal.pone.0073753. PubMed PMID: 24086292.
25. Wanner M, Lohse T, Braun J, Cabaset S, Bopp M, Krause N, et al. Occupational physical activity and all-cause and cardiovascular disease mortality: Results from two longitudinal studies in Switzerland. American journal of industrial medicine. 2019;62(7):559-67. PubMed PMID: rayyan-63657160.
26. Yu S, Yarnell JW, Sweetnam PM, Murray L. What level of physical activity protects against premature cardiovascular death? The Caerphilly study. Heart. 2003;89(5):502-6. Epub 2003/04/16. doi: 10.1136/heart.89.5.502. PubMed PMID: 12695452; PubMed Central PMCID: PMCPMC1767647.
27. Holtermann A, Hansen JV, Burr H, Sogaard K, Sjogaard G. The health paradox of occupational and leisure-time physical activity. British journal of sports medicine. 2012;46(4):291-5. Epub 2011/04/05. doi: 10.1136/bjsm.2010.079582. PubMed PMID: 21459873.
28. Holtermann A, Krause N, van der Beek AJ, Straker L. The physical activity paradox: six reasons why occupational physical activity (OPA) does not confer the cardiovascular health benefits that

leisure time physical activity does. British journal of sports medicine. 2018;52(3):149-50. Epub 2017/08/12. doi: 10.1136/bjsports-2017-097965. PubMed PMID: 28798040.
29. Tüchsen F, Endahl LA. Increasing inequality in ischaemic heart disease morbidity among employed men in Denmark 1981-1993: the need for a new preventive policy. International journal of epidemiology. 1999;28(4):640-4. Epub 1999/09/10. doi: 10.1093/ije/28.4.640. PubMed PMID: 10480690.
30. Mackenbach JP, Bos V, Andersen O, Cardano M, Costa G, Harding S, et al. Widening socioeconomic inequalities in mortality in six Western European countries. International journal of epidemiology. 2003;32(5):830-7. Epub 2003/10/16. doi: 10.1093/ije/dyg209. PubMed PMID: 14559760.
31. Coenen P, Huysmans MA, Holtermann A, Krause N, van Mechelen W, Straker LM, et al. Do highly physically active workers die early? A systematic review with meta-analysis of data from 193 696 participants. British journal of sports medicine. 2018;52(20):1320-6. Epub 2018/05/16. doi: 10.1136/bjsports-2017-098540. PubMed PMID: 29760168.
